# Supplementary material for: C-terminal amides mark proteins for degradation via SCF–FBXO31
Source: Nature. 2025 Jan 29;638(8050):519–27. doi: 10.1038/s41586-024-08475-w (PMC11821526; doi:10.1038/s41586-024-08475-w)
Supplement: Supplementary file 1 — Supplementary Fig. 1 (uncropped gels and blots), Supplementary Fig. 2 (gating strategies for flow cytometry and cell sorting) and Supplementary Methods (general synthetic procedures and characterization of compounds). [file 41586_2024_8475_MOESM1_ESM.docx]

Supplementary File 1

## Table of contents

[Table of contents 1](#_Toc181725268)

[Supplementary Figures 2](#_Toc181725269)

[General Synthetic procedures 6](#_Toc181725270)

[Peptide and protein synthesis methods 6](#_Toc181725271)

[Individual QC of synthesis products 10](#_Toc181725272)

[Characterization of fluorescein-conjugated SPPS products 10](#_Toc181725273)

[Characterization of SPPS products for sortase reaction 37](#_Toc181725274)

[Characterization of protein-peptide conjugates 48](#_Toc181725275)

[NMR spectra 55](#_Toc181725276)

## Supplementary Figures

**
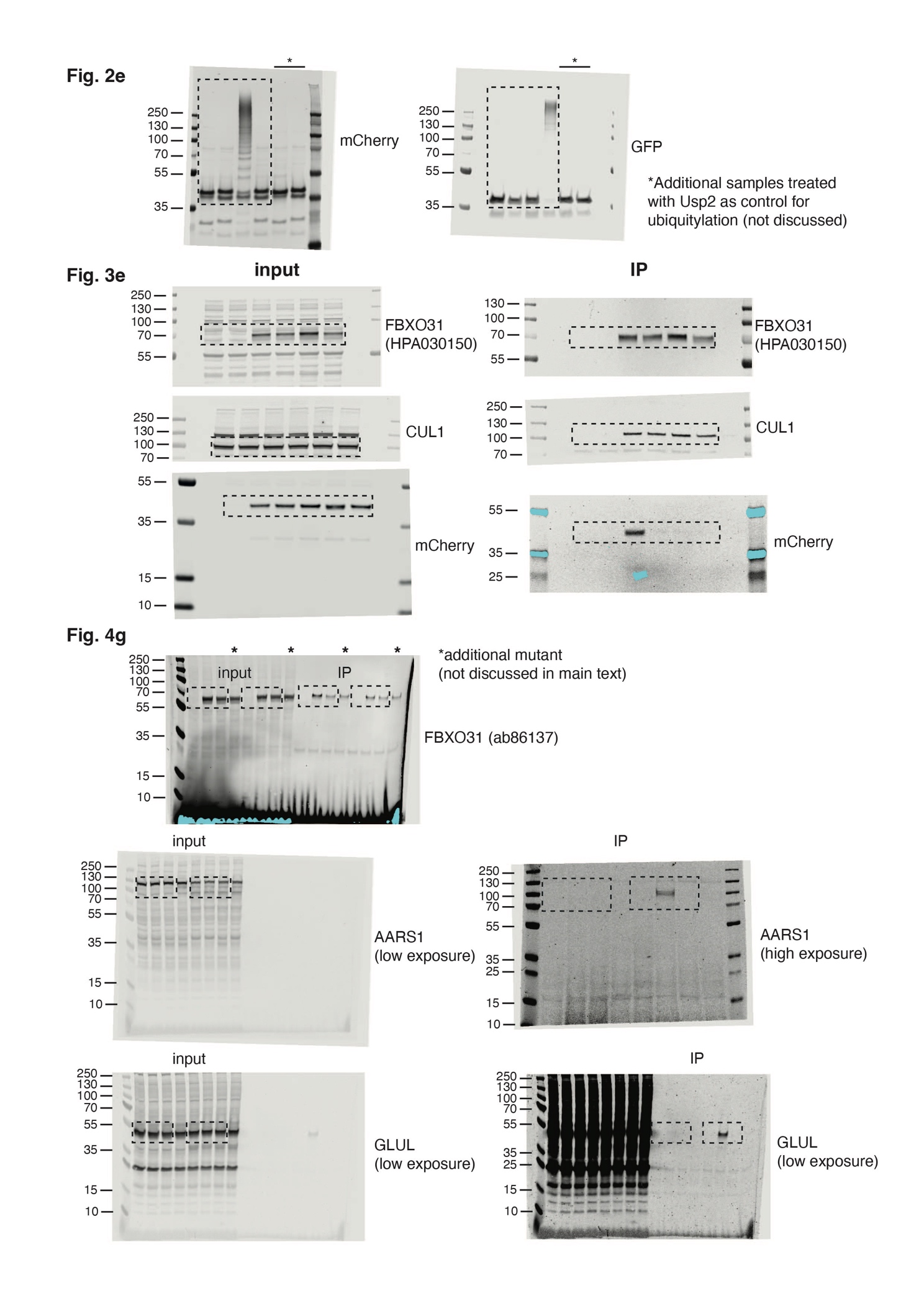
**

**Supplementary Figure 1 – Uncropped blots and gel scans**

**
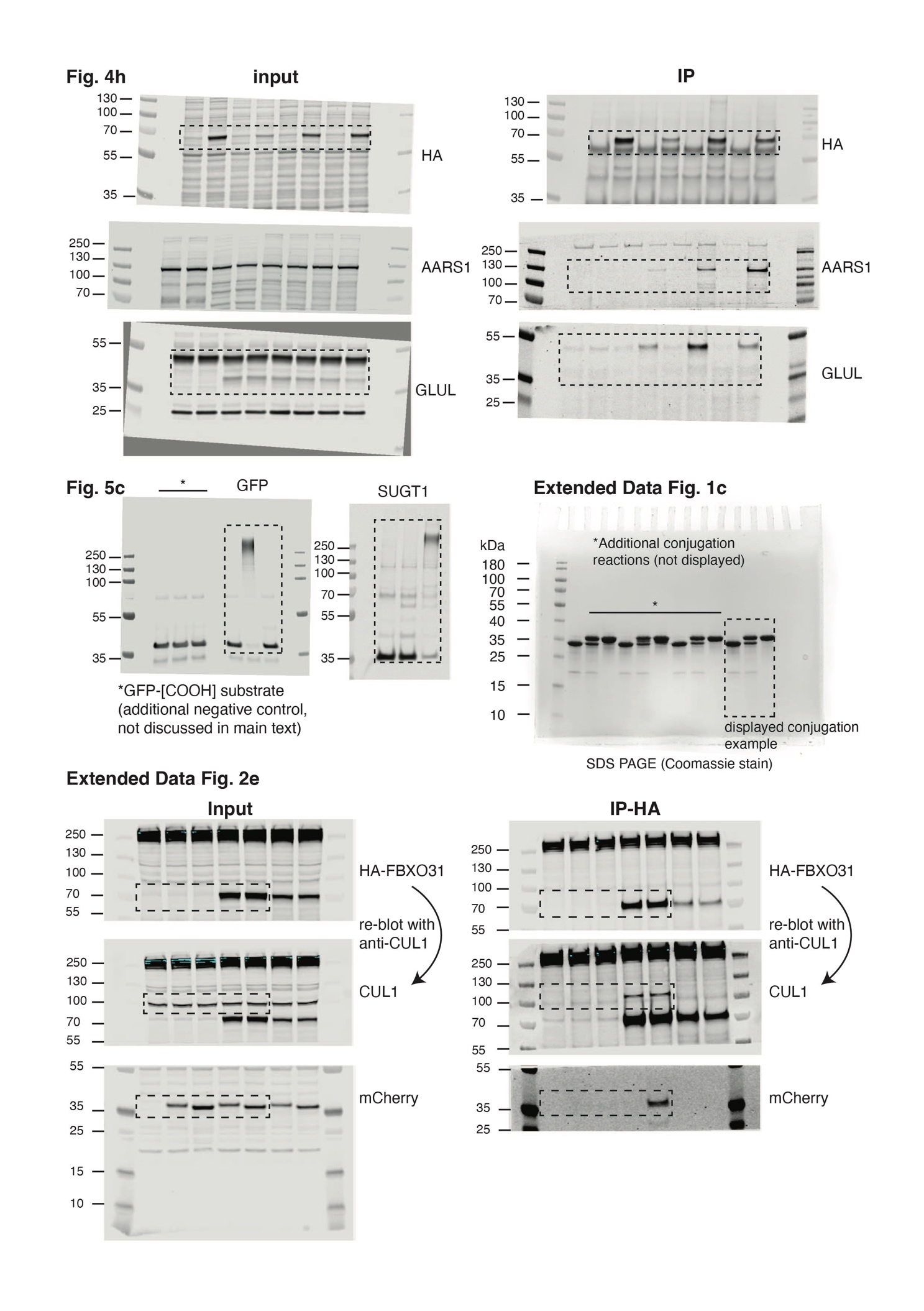
**

**Supplementary Figure 1 (continued) – Uncropped blots and gel scans**

**
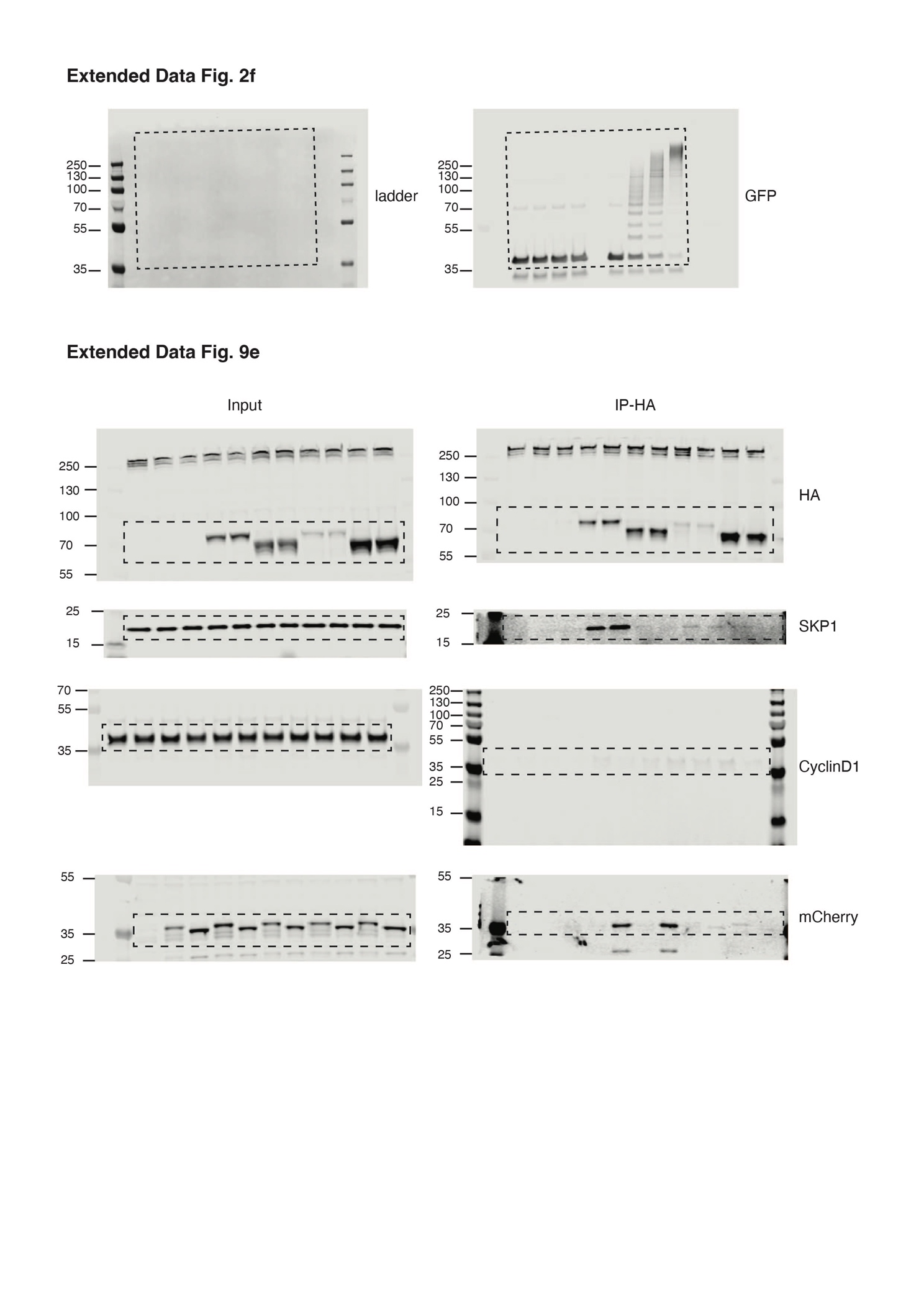
**

**Supplementary Figure 1 (continued) – Uncropped blots and gel scans**

**
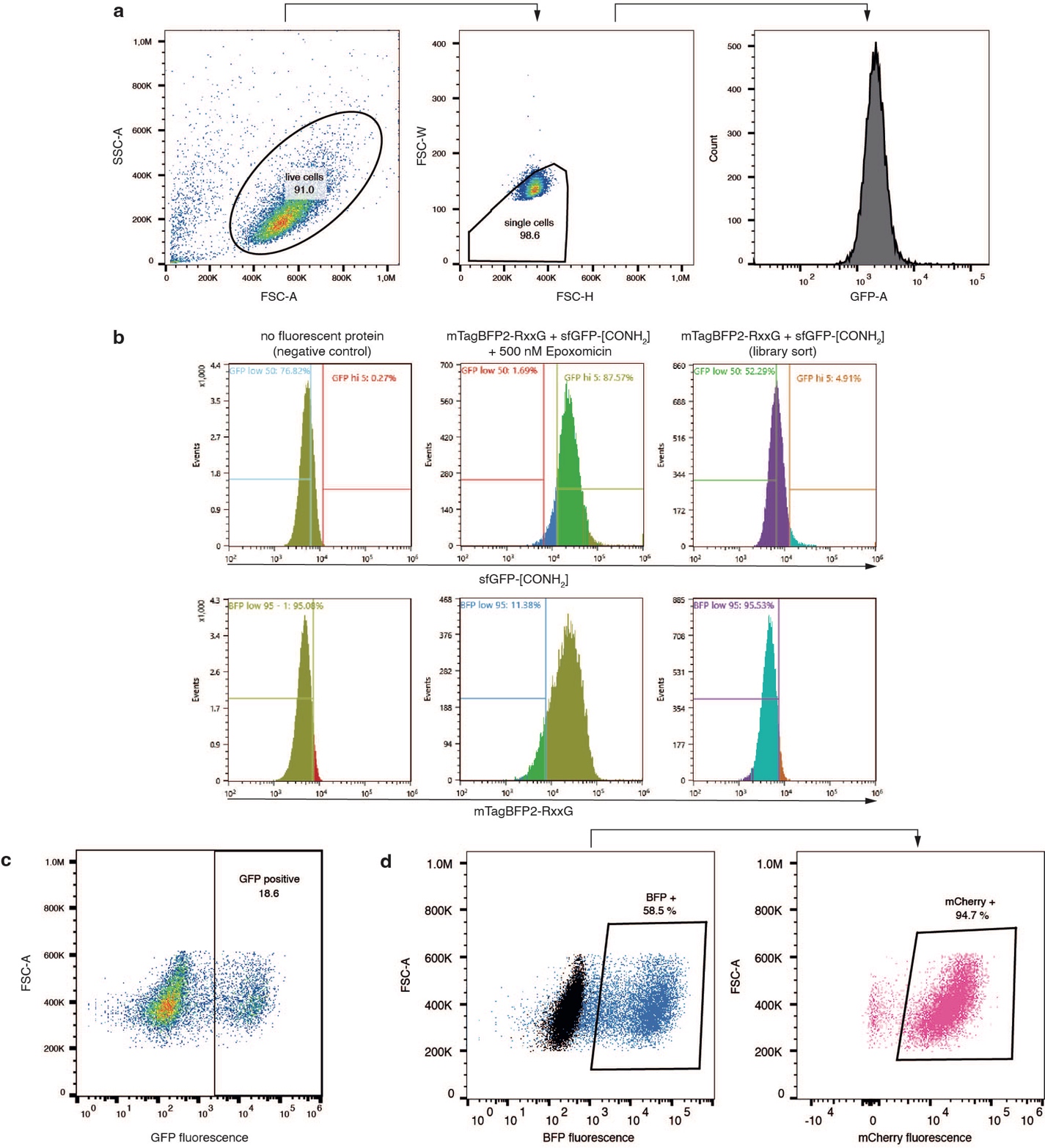
**

**Supplementary Fig. 2: Gating strategies for flow cytometry and cell sorting. a** Representative plots showing the gating strategy for measuring reporter protein levels in K562 cells. Total events were gated to include live cells and exclude duplets before extracting mean GFP fluorescence values. **b** Gating strategy used for isolating CTAP-clearance deficient cells (BFP low & GFP high) and a CTAP-proficient background population following live-cell gating and doublet exclusion as in **a**. Set-up control samples received no fluorescent reporter proteins (negative control) or BPF-RxxG and GFP-CONH_2_ followed by proteasome inhibition (500 nM epoxomicin) to assess fluorescence levels of cells deficient for reporter degradation. Gates were manually adjusted during the sort to maintain initial precentages. **c** Gating strategy used for quantifying cDNA overexpressing cells (GFP+) in competitive growth assays following live-cell gating and doublet exclusion as in **a**. **d** Gating strategy for isolating cells expressing FBXO31 cDNAs or negative control BFP (BFP +) and protein stability reporter vectors (mCherry+) for neo-substrate reporter assays.

## General Synthetic procedures

### Peptide and protein synthesis methods

Peptide synthesis reagents and solvents

Fmoc-amino acids with suitable side-chain protecting groups, HATU (1-[bis(dimethylamino)methylene]-1H-1,2,3-triazolo[4,5-b]pyridinium 3-oxide hexafluorophosphate) were purchased from Peptides International (Louisville, KY, USA), ChemImpex (Wood Dale, IL, USA) and Merck Milipore. HPLC grade CH_3_CN from Sigma-Aldrich was used for analytical and preparative HPLC purification. Trifluoroacetic acid for HPLC analytical and preparative HPLC purification was purchased from ABCR. DMF (> 99.8%) from Sigma-Aldrich and N-methylpyrrolidine from ABCR were directly used without further purification for solid phase peptide synthesis. Other commercially available reagents and solvents were purchased from Sigma-Aldrich (Buchs, Switzerland), Acros Organics (Geel, Belgium) and TCI Europe (Zwijndrecht, Belgium).

Peptide and protein conjugate characterization

High-resolution mass spectra were recorded by the Molecular and Biomolecular Analysis Service (MoBiAS) at ETH Zurich with a Bruker maXis instrument (ESI-MS measurements) equipped with an ESI source and a Q-TOF detector. Reaction monitoring was performed on a Bruker microFLEX instrument (MALDI-TOF) using 4-hydroxy-α-cyanocinnamic acid as matrix. Advanced raw data processing for bioanalytical interpretations were performed using PEAKS Studio software (Bioinformatics Solutions Inc., Canada).

Peptide purification

Peptides were analyzed and purified by reverse phase high performance liquid chromatography (RP-HPLC) on JASCO analytical and preparative instruments equipped with dual pump, mixed and in-line degasser, a variable wavelength UV detector (simultaneous detection of the eluent at 220 nm, 254 nm and 301 nm) and a Rheodyne injector with a 200 µL or 10 mL injection loop. Columns were heated to 60 ºC using a Jetstream 2 column heater (analytical) or a H_2_O water bath (preparative). The mobile-phase for RP-HPLC was Milipore-H_2_O containing 0.1% (v/v) TFA and HPLC grade CH_3_N containing 0.1% (v/v) TFA. Analytical HPLC was performed on Shiseido Capcell Pak C18 (5 μm, 4.6 mm I.D. x 250 mm) columns at a flow rate of 1 mL/min. Preparative HPLC was performed on Shiseido Capcell Pak MGIII (5 μm, 20 mm I.D. x 250 mm) at a flow rate of 40 mL/min.

General *analytical* HPLC methods:

- flow 1 mL/min, isocratic 10% CH_3_CN for 3 min, then gradient from 10% to 95% CH_3_CN in 14 min

General *preparative* HPLC methods:

- flow 40 mL/min, isocratic 5% CH_3_CN for 5 min, then gradient from 10% to 65%

CH_3_CN in 28 min.

Solid phase peptide synthesis

Loading of amino acids on solid support was performed as followed:

- Chloro-trityl resin: The amino acid (1.20 equiv of desired loading) was dissolved in CH_2_Cl_2_ (200 mM). NMM (2 equiv) was added to the solution. The solution was given to preswollen chlorotrityl resin and shaken for 1h. The resin was washed with CH_2_Cl_2_ and DMF. Remaining chloro-trityl moieties were capped with CH_2_Cl_2_/MeOH/NMM (17:2:1, v:v:v) for 1 min. The capping step was repeated once. The resin was washed with CH_2_Cl_2_ and DMF. The resin was dried using a N_2_ stream prior to usage.
- Rink amide resin: Fmoc-Rink amide resin was deprotected using 20 vol% piperidine in DMF for 2x5 min. The resin was washed thoroughly. Amino acid (1.2 equiv of desired loading) and HCTU (0.95 equiv of amino acid) were dissolved in DMF (200 mM). NMM (2 equiv of amino acid) was added. The solution was added to the preswollen Rink amide resin and shaken for 18 h. The resin was washed with CH_2_Cl_2_ and DMF. The resin was dried using a N_2_ stream prior to usage.

Peptides were synthesized on a Multisyntech Syro I parallel synthesizer using Fmoc-SPPS chemistry. The following Fmoc amino acids with side-chain protection groups were used: Fmoc-Ala-OH, Fmoc-Arg(Pbf)-OH, Fmoc-Asn(Trt)-OH, Fmoc-Asp(OtBu)-OH, Fmoc-Gln(Trt)-OH, Fmoc-Glu(OtBu)-OH, Fmoc-Gly-OH, Fmoc-His(1-Trt)-OH, Fmoc-Ile-OH, Fmoc-Leu-OH, Fmoc-Lys(Boc)-OH, Boc-Lys(Fmoc)-OH Fmoc-Met-OH, Fmoc-Phe-OH, Fmoc-Pro-OH, Fmoc-Ser(tBu)-OH, Fmoc-Thr(tBu)-OH, Fmoc-Trp(Boc)-OH, Fmoc-Tyr(tBu)-OH, Fmoc-Val-OH.

General methods on Multisyntech Syro I parallel synthesizer:

- Amino acids were dissolved in DMF to a concentration of 0.5 M. HATU was dissolved in DMF to a concentration of 0.5 M. DIPEA was dissolved in NMP to a concentration of 2 M. Amino acid, HATU and DIPEA are mixed to a final concentration of 0.2 M, 0.2 M and 0.4 M, respectively, and added to the resin. The resin was agitated for 45 min. Coupling steps were repeated once.
- Capping was performed with acetic anhydride. 20 vol% acetic anhydride in DMF was mixed with 2 M DIPEA at a ratio of 3:2 and added to the resin. The resin was agitated for 5 min. The capping step was repeated once.
- Fmoc deprotection was performed with 20 vol% piperidine in DMF for 10 min. The deprotection step was repeated once.

Preparation of Fmoc-arginol (Pbf)

Fmoc-Arg(Pbf)-OH (2.00 g, 3.10 mmol) was dissolved in dimethoxyethane (20 mL) and cooled to –15 ºC using an ice-salt bath under inert atmosphere. N-methyl morpholine (440 µL, 3.40 mmol) was added followed by isobutyl-chloroformate (373 µL, 3.40 mmol) and stirred for 30 min. After 30 min the reaction was filtered and the filtrate was immediately cooled to –15 ºC using an ice-salt bath under inert atmosphere. NaBH_4_ (350 mg, 9.25 mmol) was added in one portion and stirred for 15 min. The reaction was quenched by addition of saturated aqueous NaHCO_3_. The mixture was extracted with EtOAc (3x). The organics were washed with 0.5 M HCl, brine, dried using MgSO_4_ and concentrated to provide Fmoc-arginol-(Pbf) (1.9 g) as a white foam. The product was used without further purification.

HRMS (ESI): calculated for [C_34_H_43_N_4_O_6_S]^+^: m/z 635.2898, found: m/z 635.2890

**^1^H-NMR** (500 MHz, CDCl_3_) d 7.73 (2H, d), 7.57 (2H, d), 7.37 (2H, t), 7.25 (2H, m), 6.30 (3H, brs), 5.64 (1H, brs), 4.36 (2H, d), 4.15 (1H, t), 3.71-3.53 (3H, m), 3.23 (2H, brs), 2.91 (2H, s), 2.58 (3H, s), 2.51 (3H, s), 2.08 (3H, s), 1.67-1.49 (4H, m), 1.44 (6H, s)

**^13^C-NMR** (125 MHz, CDCl_3_) d 159.07, 157.13, 156.39, 144.01, 143.93, 141.39, 138.55, 132.46, 127.82, 127.20, 125.25, 124.89, 120.07, 117.79, 86.62, 66.84, 64.83, 47.34, 43.30, 41.18, 28.69, 25.70, 19.45, 18.10, 12.61

**Synthesis of fluorescent peptides for fluorescence polarization measurement**

General method

Amino acids were loaded on chlorotrityl resin to access C-terminal carboxylic acids or Rink amide to access C-terminal amides according to the general methods. Automated peptide elongation was carried out on a Multisyntech Syro I parallel synthesizer according to the general peptide methods. N-terminal Boc-Lysine(Fmoc)-OH was coupled manually (2 equiv, 90 min). Fmoc group was removed by treatment with 20 vol% piperidine. The resin was washed thoroughly. All following steps were performed in the dark. FITC (3 equiv) and NMM (6 equiv) were dissolved in DMF, given to the resin and shaken for 2 h. The peptide was cleaved from the resin using TFA/DODT/H_2_O (95:2.5:2.5, v/v) for 1 h. The resin was removed by filtration and the filtrate concentrated under reduced pressure. The solution was triturated with Et_2_O and centrifuged to obtain crude peptide. The crude peptide was dissolved in H_2_O/CH_3_N (1:1, v/v) + 0.1% (v/v) TFA and purified using preparative HPLC unless stated otherwise.

Preparation of peptide alcohol

Fmoc-arginol (Pbf) (190 mg, 0.3 mmol) in 2 vol% DBU in DMF was added to 2-chlorotrityl chloride resin (0.1 mmol) and shaken overnight. The resin was washed with DMF and CH_2_Cl_2_ and capped according to the general procedure. Fmoc-Leu (212 mg, 0.6 mmol) was dissolved in DMF/CH_2_Cl_2_ (1:1, v/v) at 0 ºC. DIC (47 µL, 0.3 mmol) and DMAP (3 mg) were added and then given to the resin. The resin was shaken for 6 h and the coupling was repeated two additional times. Following standard Fmoc-SPPS, Boc-Lys(Fmoc)-OH and FITC were coupled as described above. Following resin cleavage, the crude peptide was dissolved in PBS 1 M NaHCO_3_ pH 8.0 was added and the pH adjusted to 8.0. The solution as incubated for 2 h at room temperature, acidified using TFA and purified by preparative RP-HPLC.

- - 1. **Preparation of peptide methyl-ester**

Preparation of peptide methyl-ester

Cyanosulfurylide resin was prepared according to previously reported procedures and Fmoc-Arg(Pbf)-OH was loaded. Following standard Fmoc-SPPS, Boc-Lys(Fmoc)-OH and FITC were coupled as described above. The resin was thoroughly washed with DMF and CH_2_Cl_2_ and dried. The resin was resuspended in THF/MeOH (1:1, v/v) and N-chlorosuccinimide (40 mg) was added and the mixture incubated for 20 min. The filtrate was removed, and the resin washed with THF. The filtrate was concentrated and standard cleavage cocktail was added. The solution was triturated with Et_2_O and centrifuged to obtain crude peptide. The crude peptide was dissolved in H_2_O/CH_3_N (1:1, v/v) + 0.1% (v/v) TFA and purified using preparative HPLC.

**Peptide library synthesis**

Isokinetic amino acid mixture

Ala (3.4 mol%), Arg (6.5 mol%), Asn (5.3 mol%), Asp (3.5 mol%), Gln (5.3 mol%), Glu (3.6 mol%), Gly (2.9 mol%), His (3.5 mol%), Ile (17.4 mol%), Leu (4.9 mol%), Lys (6.2 mol%), Met (3.8 mol%), Phe (2.5 mol%), Pro (4.3 mol%), Ser (2.8 mol%), Thr (4.8 mol%), Trp (3.8 mol%), Tyr (4.1 mol%), Val (11.3 mol%)

Amino acids were dissolved in DMF to a total concentration of 0.5 M and used as is for resin loading and peptide elongation steps.

Synthesis of three variable peptide library with C-terminal carboxylic acid

**GGGKYRYDVPDYXXX-COOH**

Resin loading and peptide synthesis was performed as described in the general methods using the isokinetic amino acid mixture for positions X. Automated peptide elongation was carried out on a Multisyntech Syro I parallel synthesizer according to the general peptide methods. The peptide was cleaved form the resin using TFA/DODT/H_2_O (95:2.5:2.5, v/v) for 1 h. The resin was removed by filtration and the filtrate concentrated under reduced pressure. The solution was triturated with Et_2_O and centrifuged to obtain crude peptide. Et_2_O was repeated three times. The crude peptide was dissolved in H_2_O/CH_3_N (1:1, v/v) and lyophilized to obtain an off-white powder.

Synthesis of three variable peptide library with C-terminal amide

**GGGKYRYDVPDYXXX-CONH_2_**

Resin loading and peptide synthesis was performed as described in the general methods using the isokinetic amino acid mixture for positions X. Automated peptide elongation was carried out on a Multisyntech Syro I parallel synthesizer according to the general peptide methods. The peptide was cleaved form the resin using TFA/DODT/H_2_O (95:2.5:2.5, v/v) for 1 h. The resin was removed by filtration and the filtrate concentrated under reduced pressure. The solution was triturated with Et_2_O and centrifuged to obtain crude peptide. Et_2_O was repeated three times. The crude peptide was dissolved in H_2_O/CH_3_N (1:1, v/v) and lyophilized to obtain an off-white powder.

## Individual QC of synthesis products

### Characterization of fluorescein-conjugated SPPS products

Synthesis of fluorescein-pep2–R–COOH

**Fluorescein–KEEDEKGSRASDDFRDLR–COOH**

The peptide was obtained as a yellow-orange solid.

HRMS (ESI): calculated for [C_108_H_154_N_30_O_40_S]^2+^: m/z 1271.5324, found: m/z 1271.5347

**
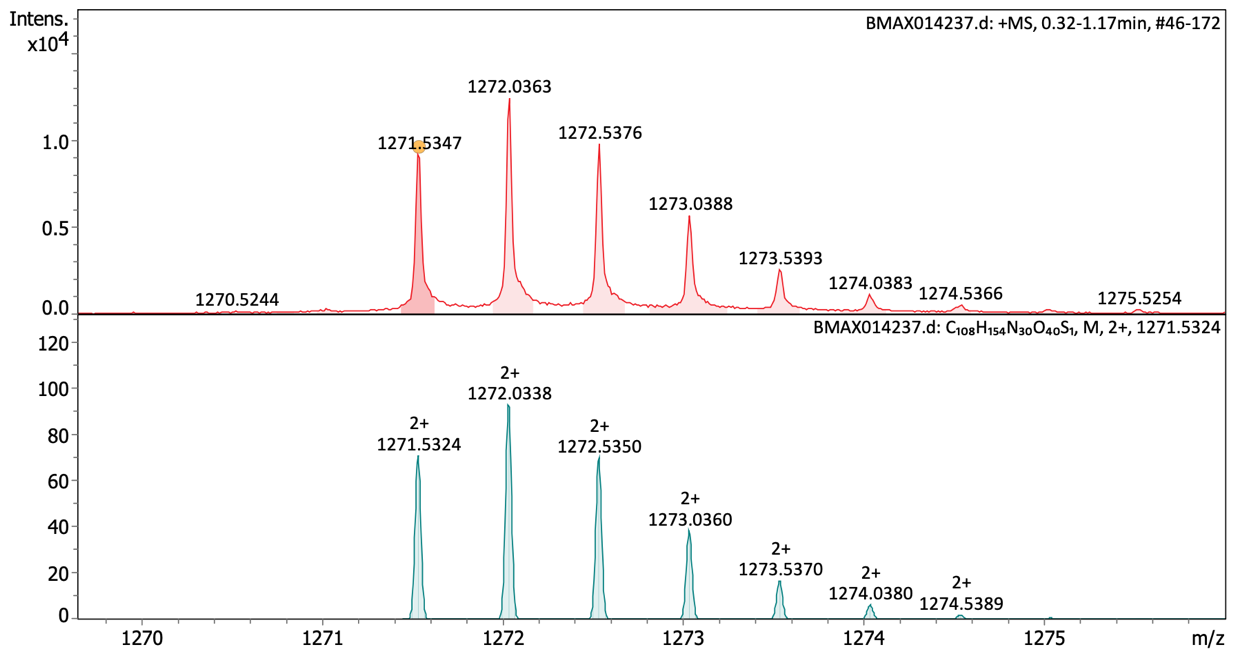
**

**Characterization of fluorescein-pep2–R–COOH** Analytical RP-HPLC of purified peptide. HRMS (ESI) spectrum of purified peptide showing recorded mass spectrum (upper panel) and calculated spectrum (lower panel).

Synthesis of fluorescein-pep2–R–CONH_2_

**Fluorescein–KEEDEKGSRASDDFRDLR–CONH_2_**

The peptide was obtained as a yellow-orange solid.

HRMS (ESI): calculated for [C_108_H_155_N_31_O_39_S]^2+^: m/z 1271.0404, found: m/z 1271.0423

**
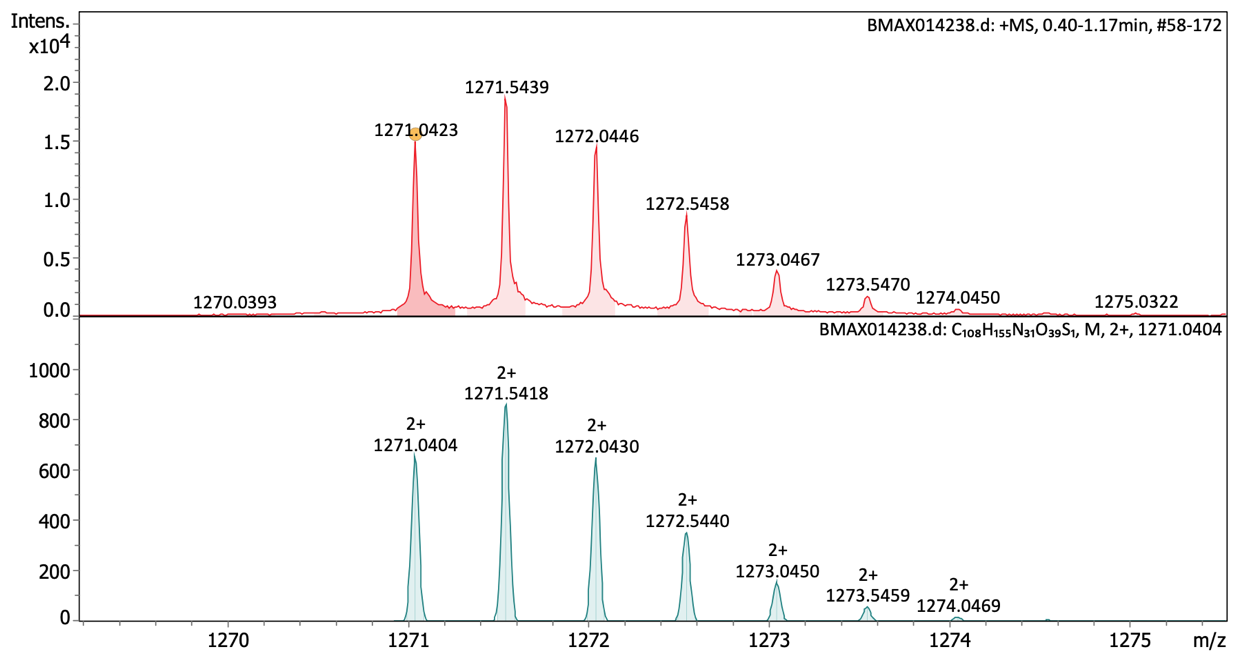
**

**Characterization of fluorescein-pep2–R–CONH_2_** Analytical RP-HPLC of purified peptide. HRMS (ESI) spectrum of purified peptide showing recorded mass spectrum (upper panel) and calculated spectrum (lower panel).

Synthesis of fluorescein-KAA-CycD1(286-295)–COOH

**Fluorescein– KAATPTDVRDVDI–COOH**

The peptide was obtained as a yellow-orange solid.

HRMS (ESI): calculated for [C_80_H_114_N_18_O_27_S]^2+^: m/z 895.3905, found: m/z 895.3916

**
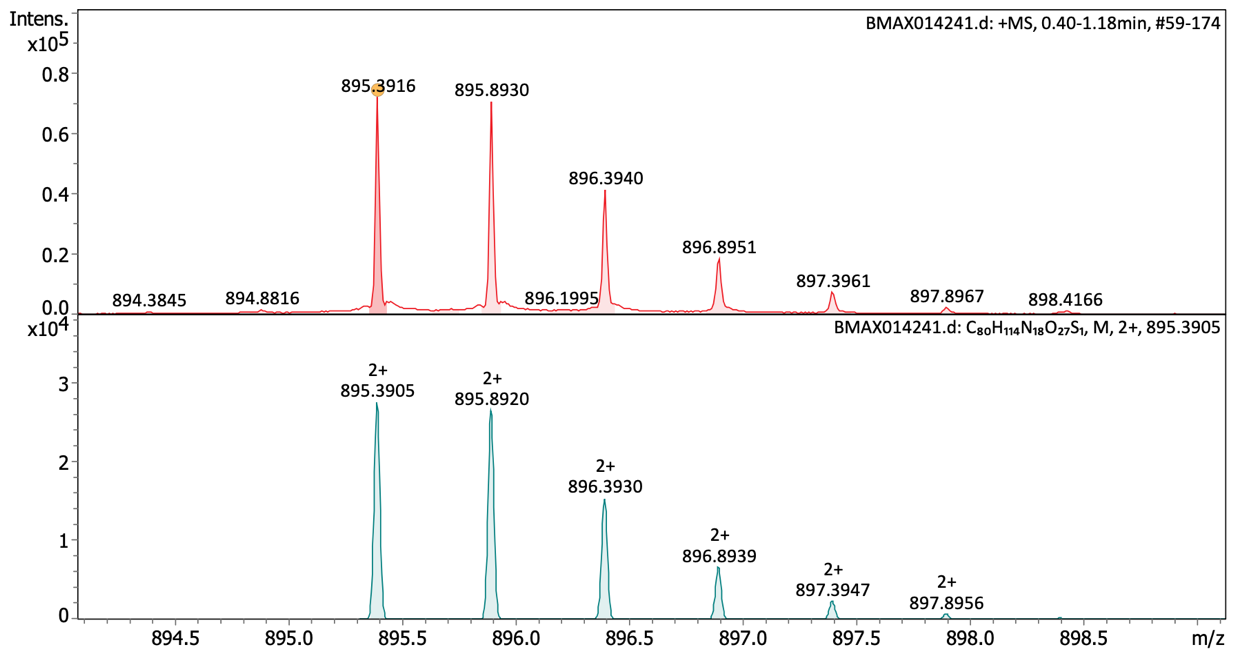
**

**Characterization of fluorescein-KAA-CycD1(286-295)–COOH** Analytical RP-HPLC of purified peptide. HRMS (ESI) spectrum of purified peptide showing recorded mass spectrum (upper panel) and calculated spectrum (lower panel).

Synthesis of fluorescein-KAA-CycD1(286-295)–CONH_2_

**Fluorescein– KAATPTDVRDVDI–CONH_2_**

The peptide was obtained as a white solid.

HRMS (ESI): calculated for [C_80_H_115_N_19_O_26_S]^2+^: m/z 894.8985, found: m/z 894.901

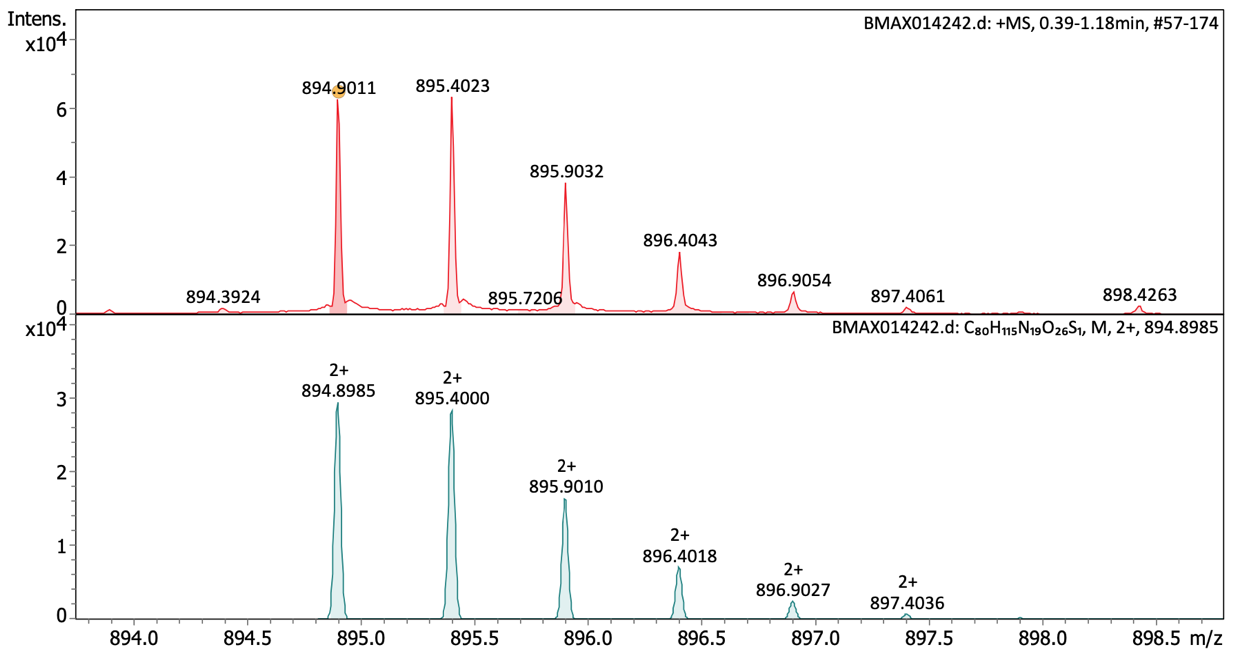


**Characterization of fluorescein-KAA-CycD1(286-295)–CONH_2_** Analytical RP-HPLC of purified peptide. HRMS (ESI) spectrum of purified peptide showing recorded mass spectrum (upper panel) and calculated spectrum (lower panel).

Synthesis of fluorescein-pep3–A–CONH_2_

**Fluorescein-KKYRYDVPDYSAA–CONH_2_**

The peptide was obtained as an orange solid and used without preparative RP-HPLC purification.

HRMS (ESI): calculated for [C_93_H_120_N_20_O_26_S_1_]^2+^: m/z 982.4196, found: m/z 982.4212


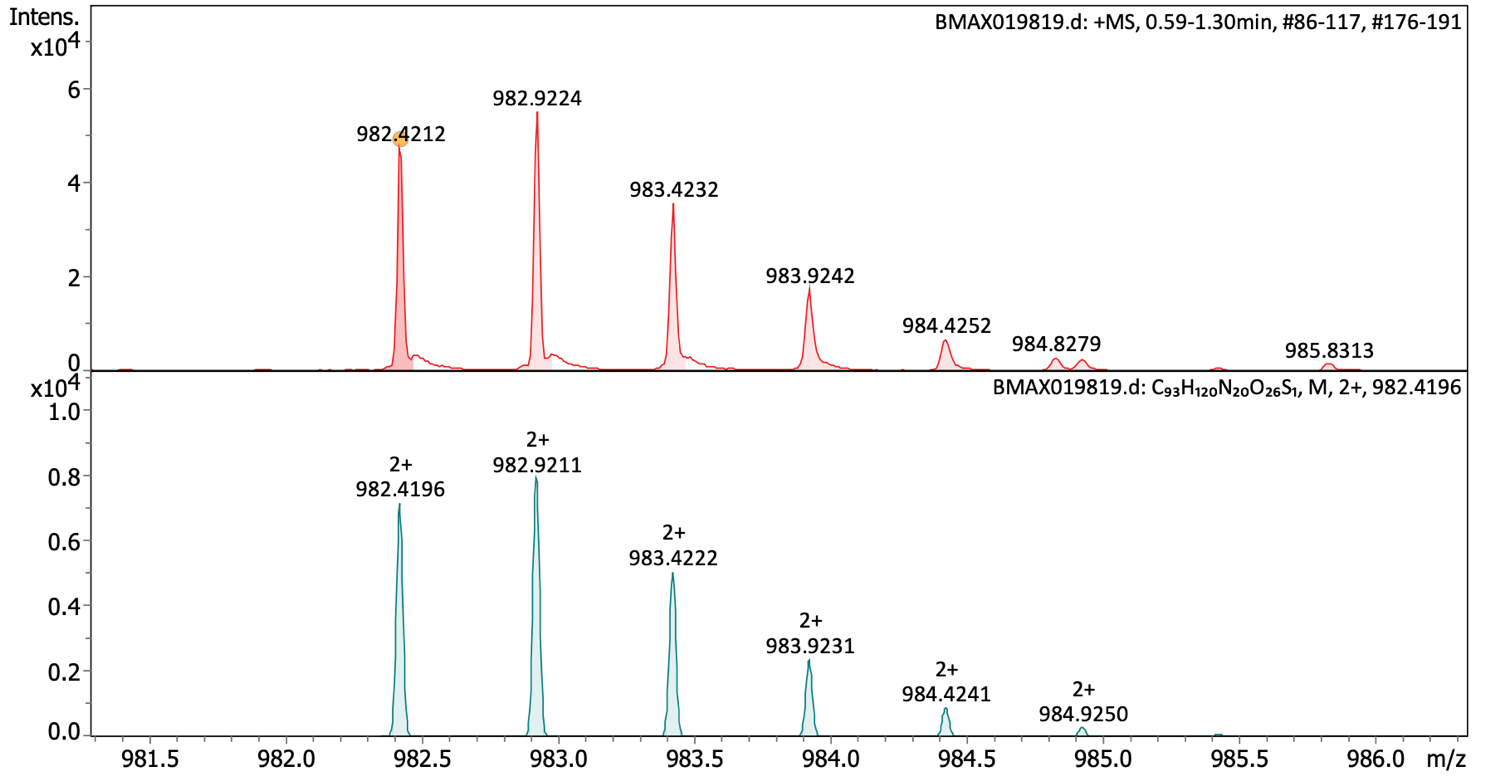


**Characterization of fluorescein-pep3–A–CONH_2_** HRMS (ESI) spectrum of purified peptide showing recorded mass spectrum (upper panel) and calculated spectrum (lower panel).

Synthesis of fluorescein-pep3–C–CONH_2_

**Fluorescein-KKYRYDVPDYSAC–CONH_2_**

The peptide was obtained as an orange solid and used without preparative RP-HPLC purification.

HRMS (ESI): calculated for [C_93_H_120_N_20_O_26_S_2_]^2+^: m/z 988.4057, found: m/z 988.4070


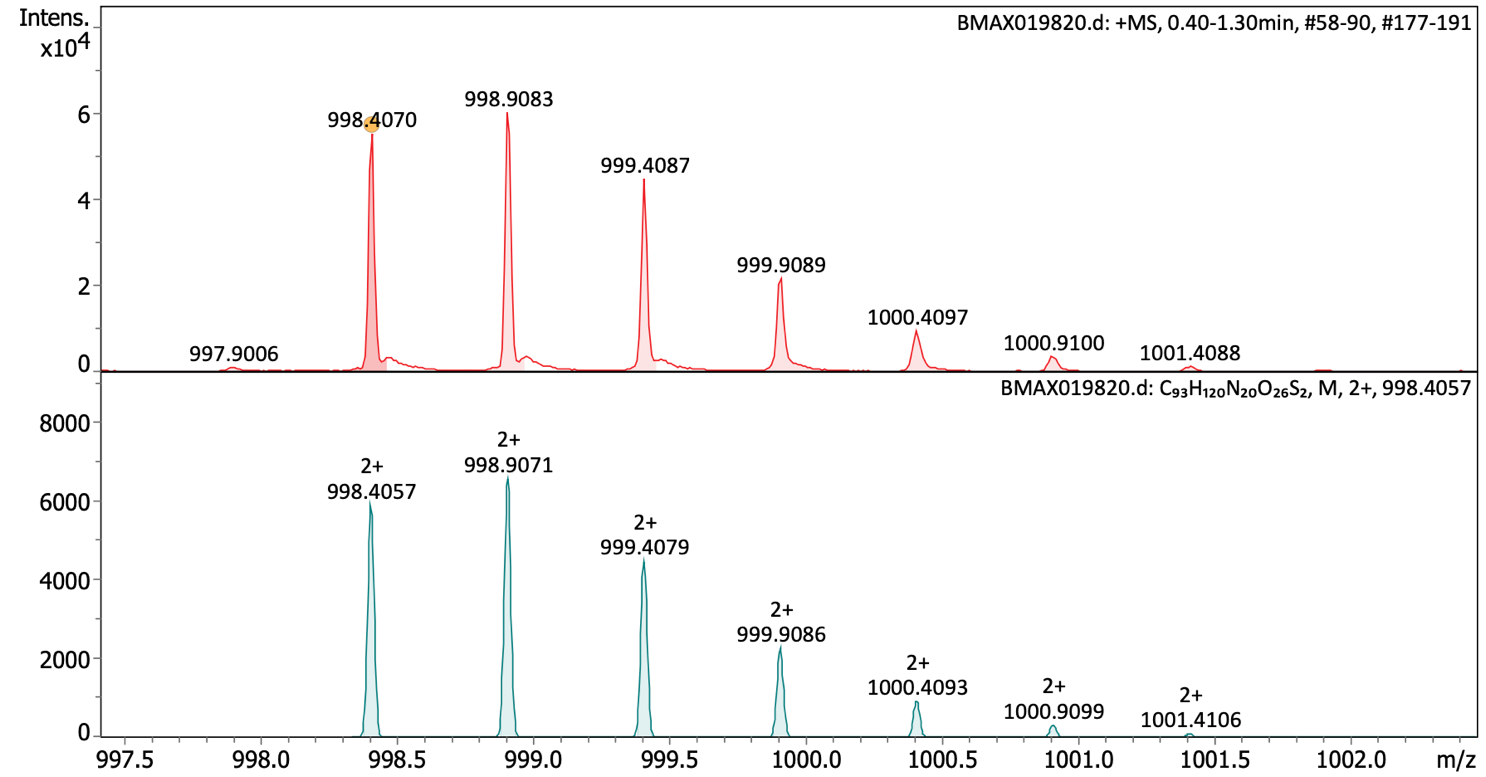


**Characterization of fluorescein-pep3–C–CONH_2_** HRMS (ESI) spectrum of purified peptide showing recorded mass spectrum (upper panel) and calculated spectrum (lower panel).

Synthesis of fluorescein-pep3–D–CONH_2_

**Fluorescein-KKYRYDVPDYSAD–CONH_2_**

The peptide was obtained as an orange solid and used without preparative RP-HPLC purification.

HRMS (ESI): calculated for [C_94_H_120_N_20_O_28_S_1_]^2+^: m/z 1004.4145, found: m/z 1004.4164


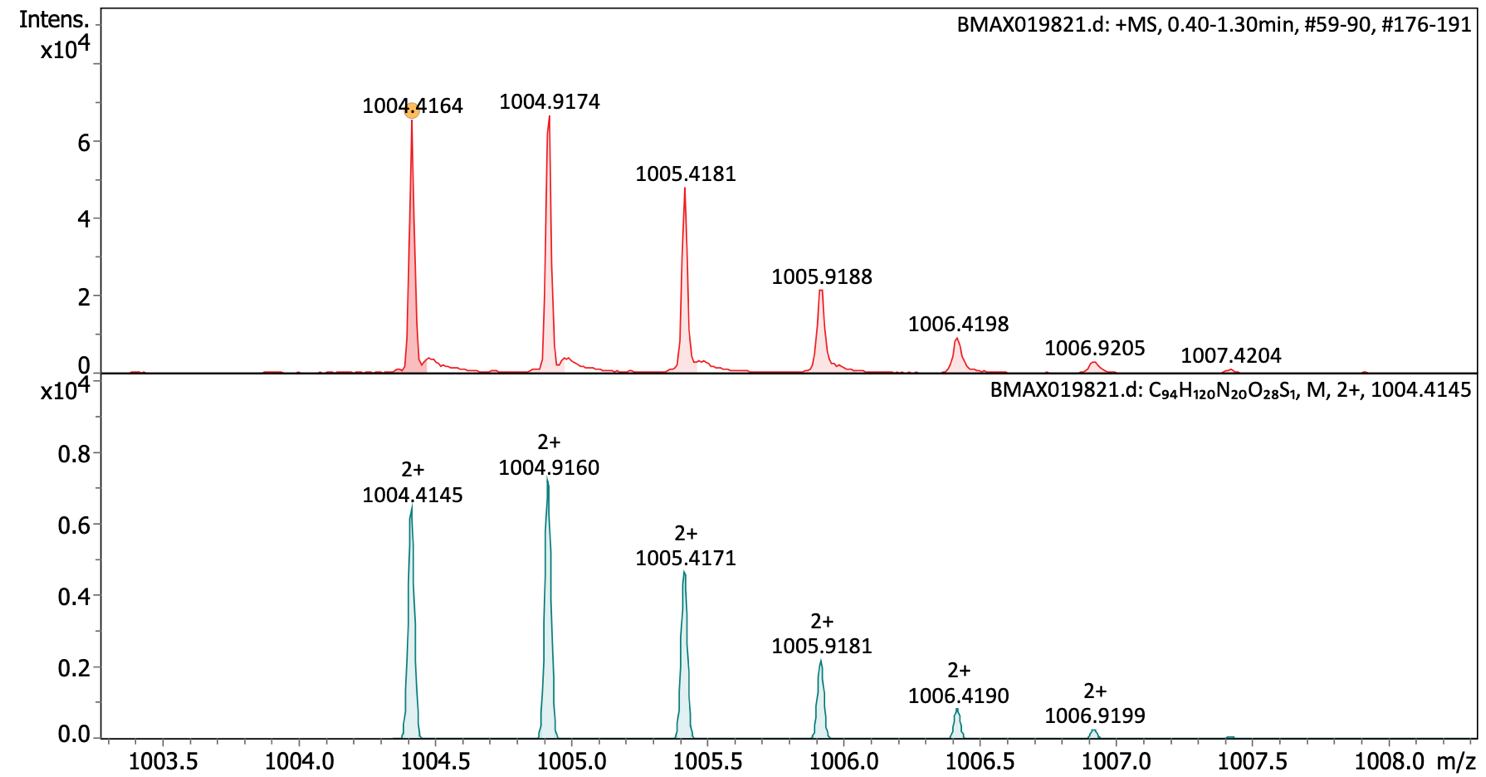


**Characterization of fluorescein-pep3–D–CONH_2_** HRMS (ESI) spectrum of purified peptide showing recorded mass spectrum (upper panel) and calculated spectrum (lower panel).

Synthesis of fluorescein-pep3–E–CONH_2_

**Fluorescein-KKYRYDVPDYSAE–CONH_2_**

The peptide was obtained as an orange solid and used without preparative RP-HPLC purification.

HRMS (ESI): calculated for [C_95_H_122_N_20_O_28_S_1_]^2+^: m/z 1011.4224, found: m/z 1011.4241


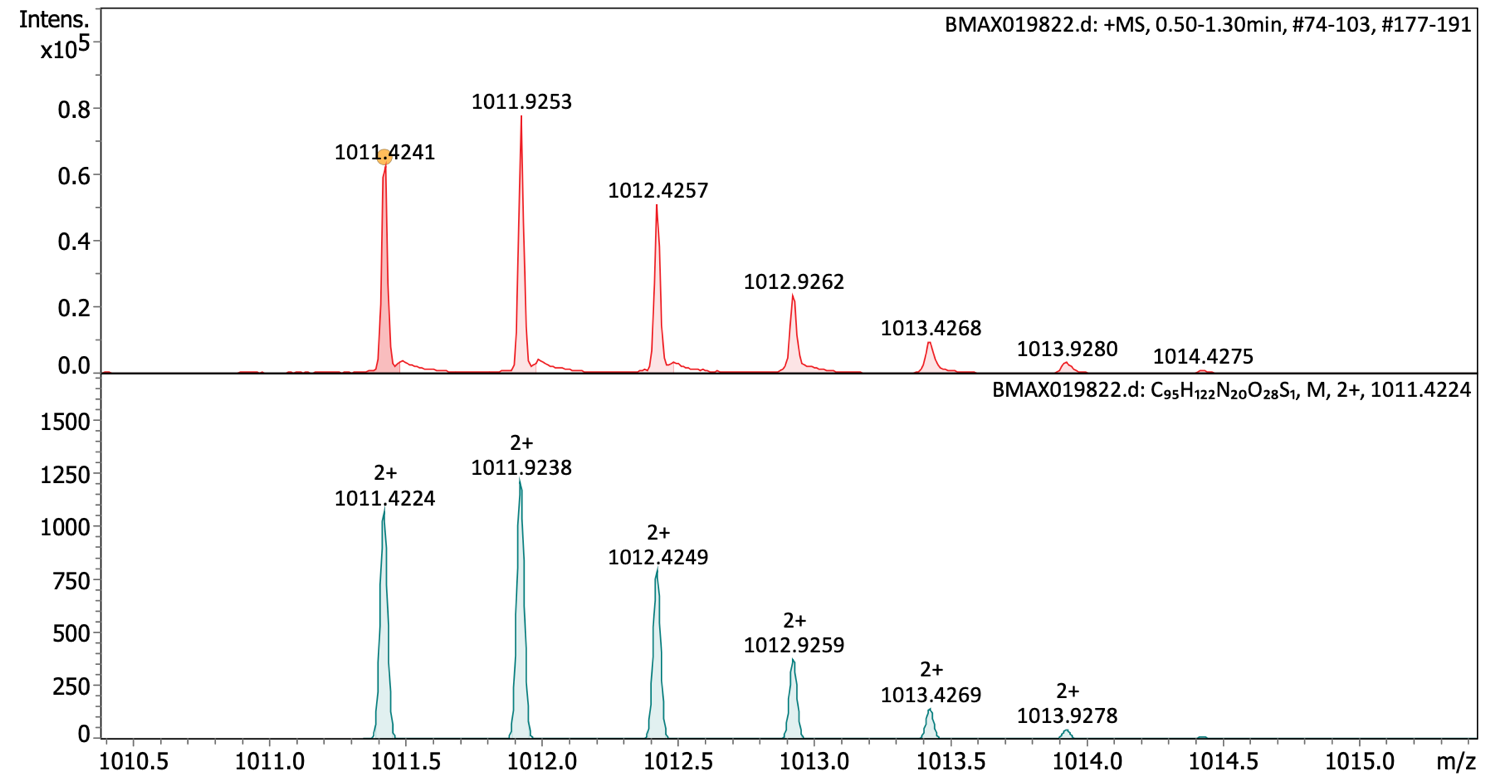


**Characterization of fluorescein-pep3–E–CONH_2_** HRMS (ESI) spectrum of purified peptide showing recorded mass spectrum (upper panel) and calculated spectrum (lower panel).

Synthesis of fluorescein-pep3–F–CONH_2_

**Fluorescein-KKYRYDVPDYSAF–CONH_2_**

The peptide was obtained as an orange solid and used without preparative RP-HPLC purification.

HRMS (ESI): calculated for [C_99_H_124_N_20_O_26_S_1_]^2+^: m/z 1020.4353, found: m/z 1020.4373


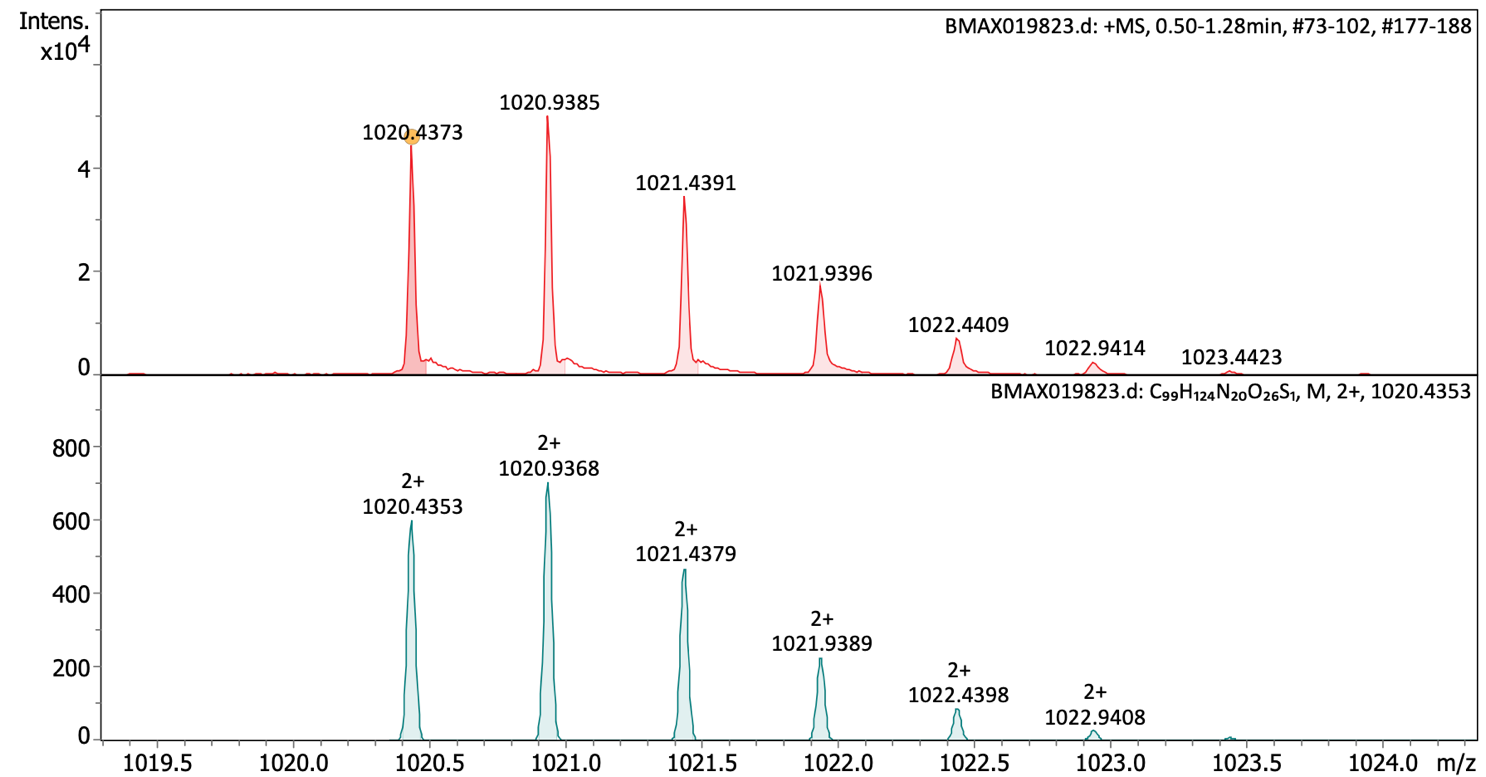


**Characterization of fluorescein-pep3–F–CONH_2_** HRMS (ESI) spectrum of purified peptide showing recorded mass spectrum (upper panel) and calculated spectrum (lower panel).

Synthesis of fluorescein-pep3–G–CONH_2_

**Fluorescein-KKYRYDVPDYSAG–CONH_2_**

The peptide was obtained as an orange solid and used without preparative RP-HPLC purification.

HRMS (ESI): calculated for [C_92_H_118_N_20_O_26_S_1_]^2+^: m/z 975.4118, found: m/z 975.4140


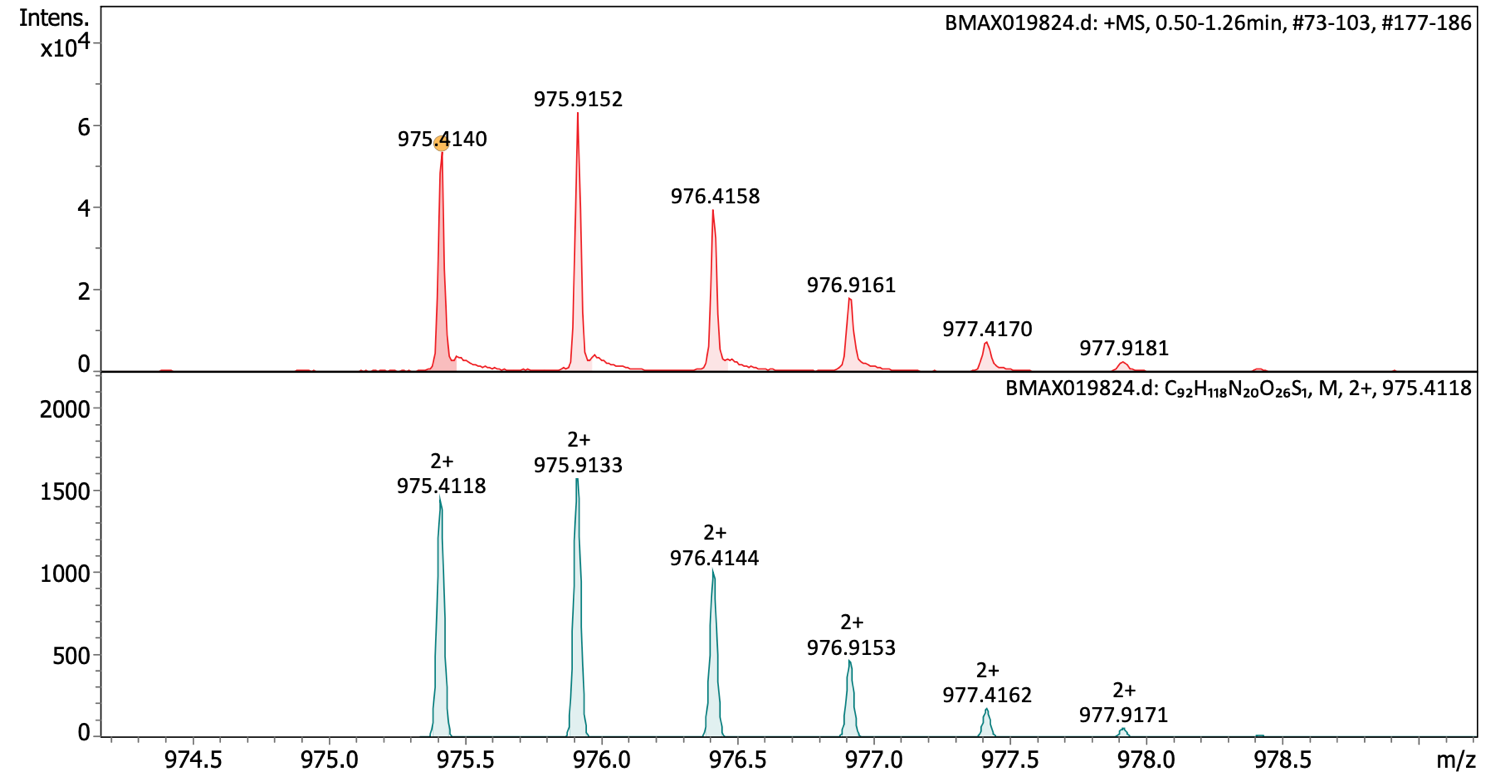


**Characterization of fluorescein-pep3–G–CONH_2_** HRMS (ESI) spectrum of purified peptide showing recorded mass spectrum (upper panel) and calculated spectrum (lower panel).

Synthesis of fluorescein-pep3–H–CONH_2_

**Fluorescein-KKYRYDVPDYSAH–CONH_2_**

The peptide was obtained as an orange solid and used without preparative RP-HPLC purification.

HRMS (ESI): calculated for [C_96_H_122_N_22_O_26_S_1_]^2+^: m/z 1015.4305, found: m/z 1015.4316


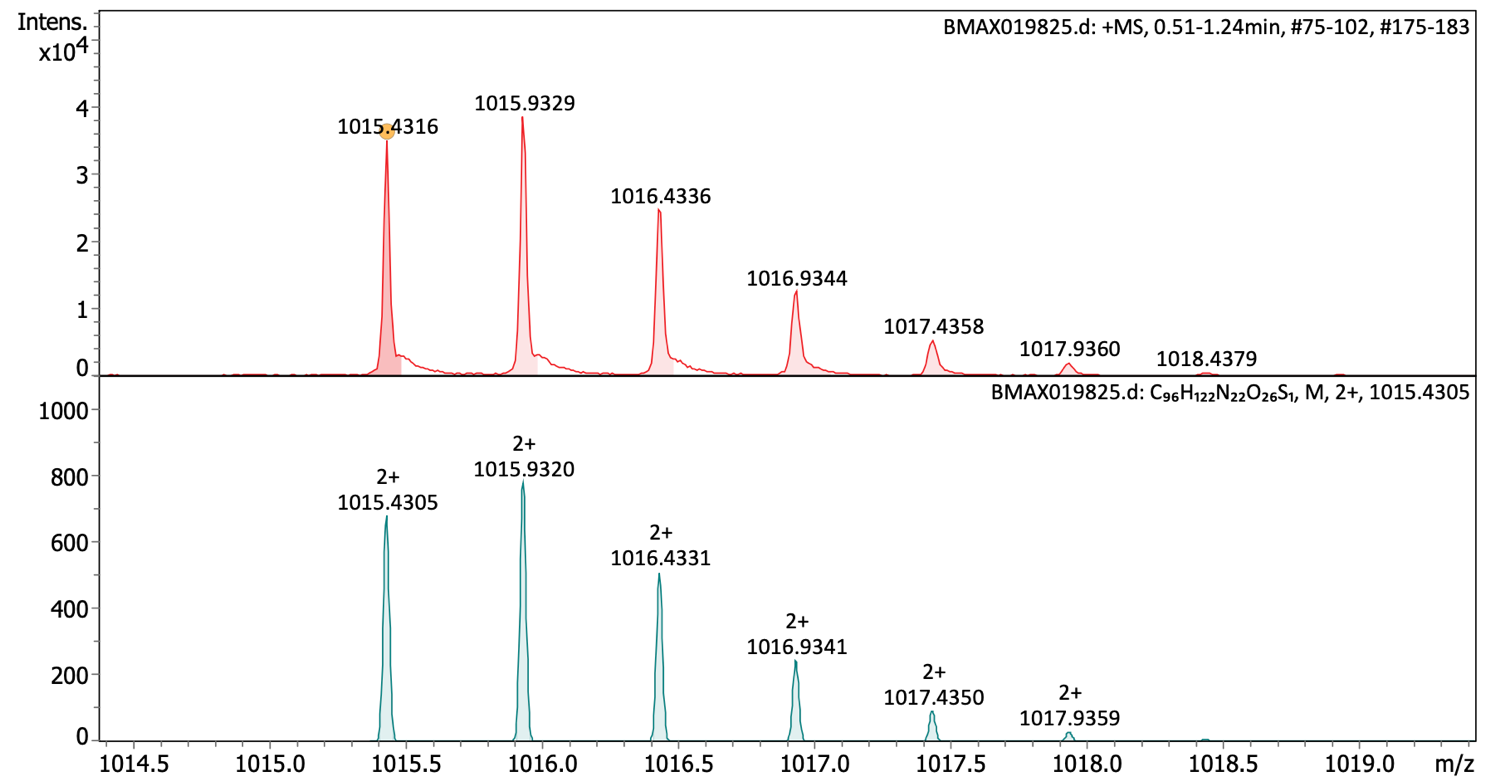


**Characterization of fluorescein-pep3–H–CONH_2_** HRMS (ESI) spectrum of purified peptide showing recorded mass spectrum (upper panel) and calculated spectrum (lower panel).

Synthesis of fluorescein-pep3–I–CONH_2_

**Fluorescein-KKYRYDVPDYSAI–CONH_2_**

The peptide was obtained as an orange solid and used without preparative RP-HPLC purification.

HRMS (ESI): calculated for [C_96_H_126_N_20_O_26_S_1_]^2+^: m/z 1003.4431, found: m/z 1003.4445


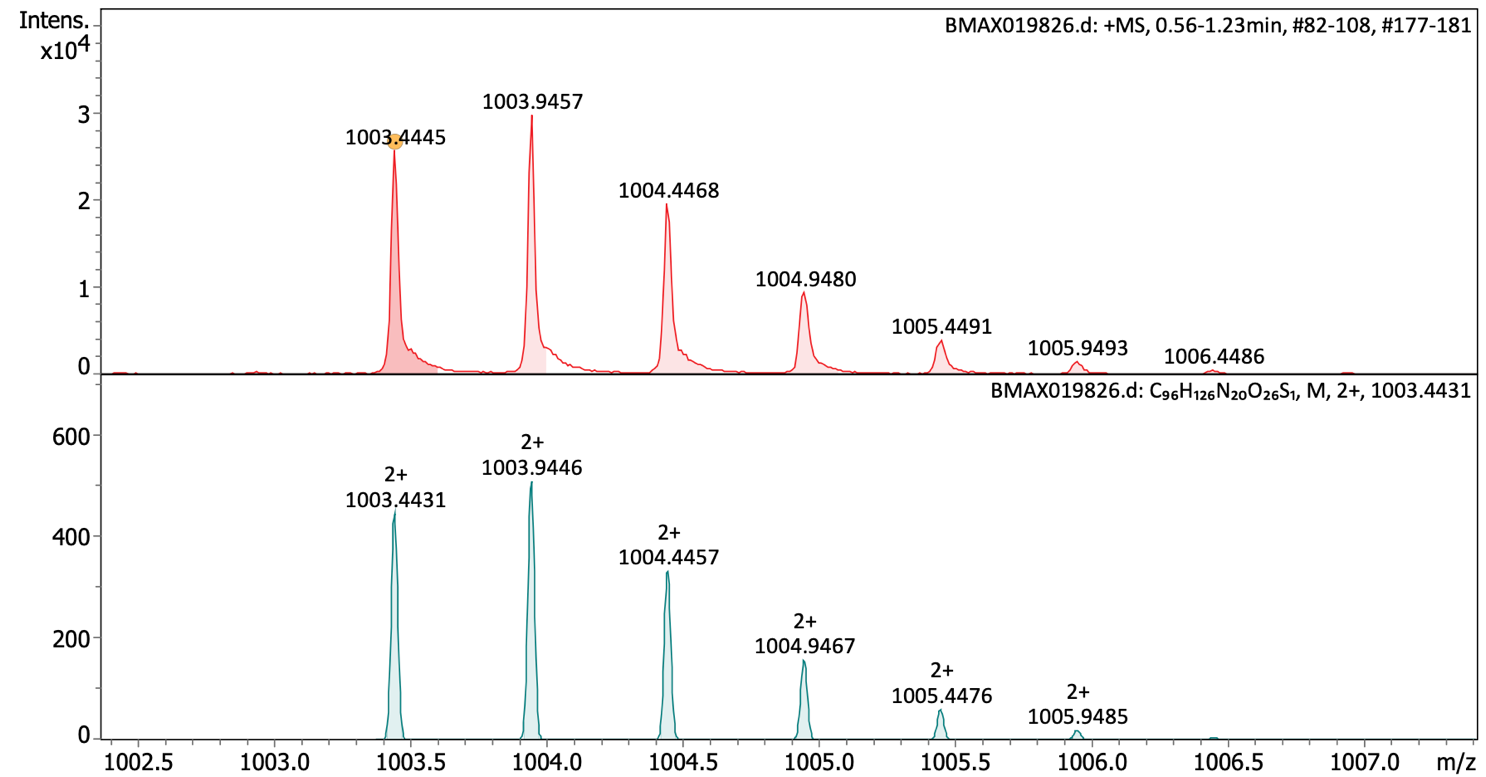


**Characterization of fluorescein-pep3–I–CONH_2_** HRMS (ESI) spectrum of purified peptide showing recorded mass spectrum (upper panel) and calculated spectrum (lower panel).

Synthesis of fluorescein-pep3–K–CONH_2_

**Fluorescein-KKYRYDVPDYSAK–CONH_2_**

The peptide was obtained as an orange solid and used without preparative RP-HPLC purification.

HRMS (ESI): calculated for [C_96_H_127_N_21_O_26_S_1_]^2+^: m/z 1010.9485, found: m/z 1010.9484


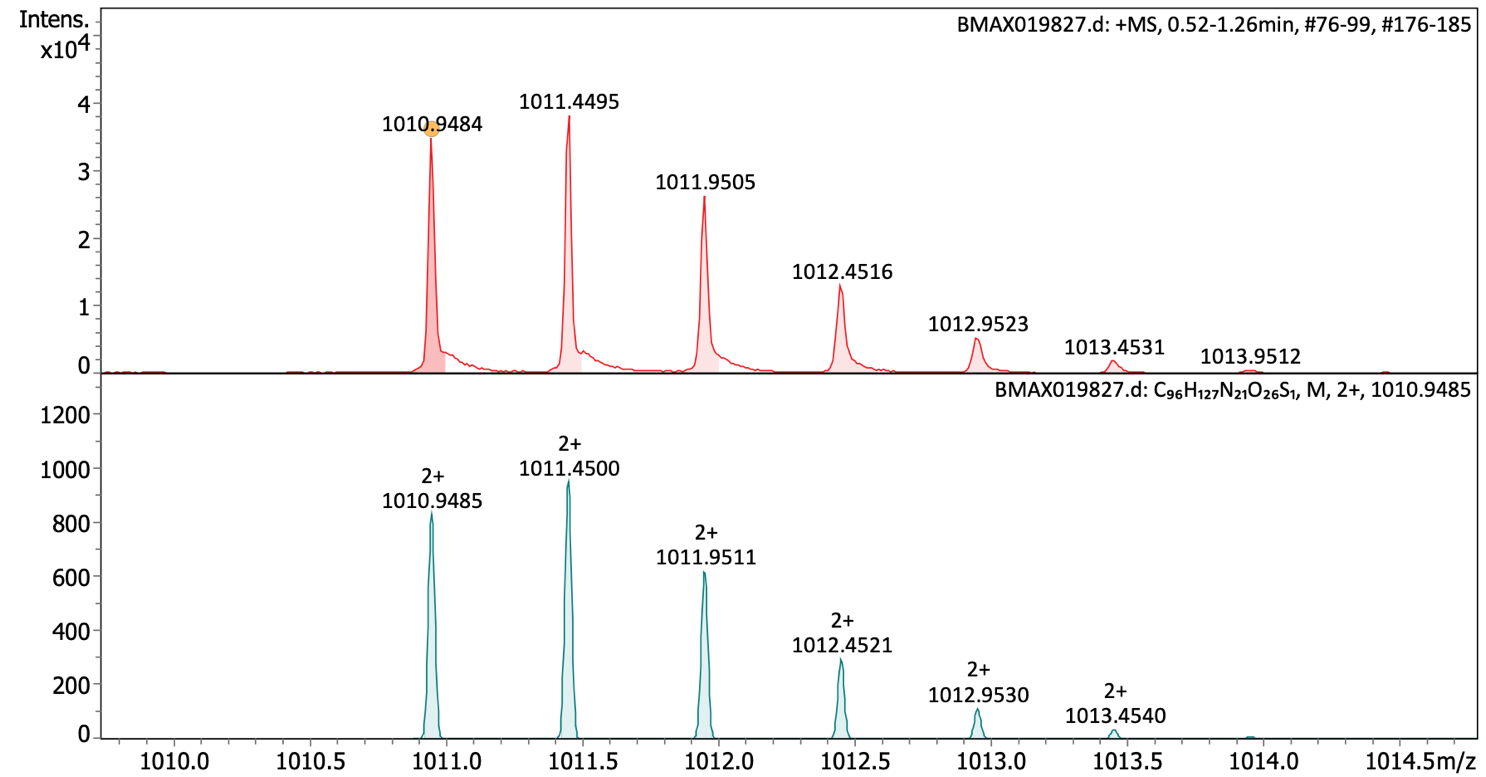


**Characterization of fluorescein-pep3–K–CONH_2_** HRMS (ESI) spectrum of purified peptide showing recorded mass spectrum (upper panel) and calculated spectrum (lower panel).

Synthesis of fluorescein-pep3–L–CONH_2_

**Fluorescein-KKYRYDVPDYSAL–CONH_2_**

The peptide was obtained as an orange solid and used without preparative RP-HPLC purification.

HRMS (ESI): calculated for [C_96_H_126_N_20_O_26_S_1_]^2+^: m/z 1003.4431, found: m/z 1003.4442


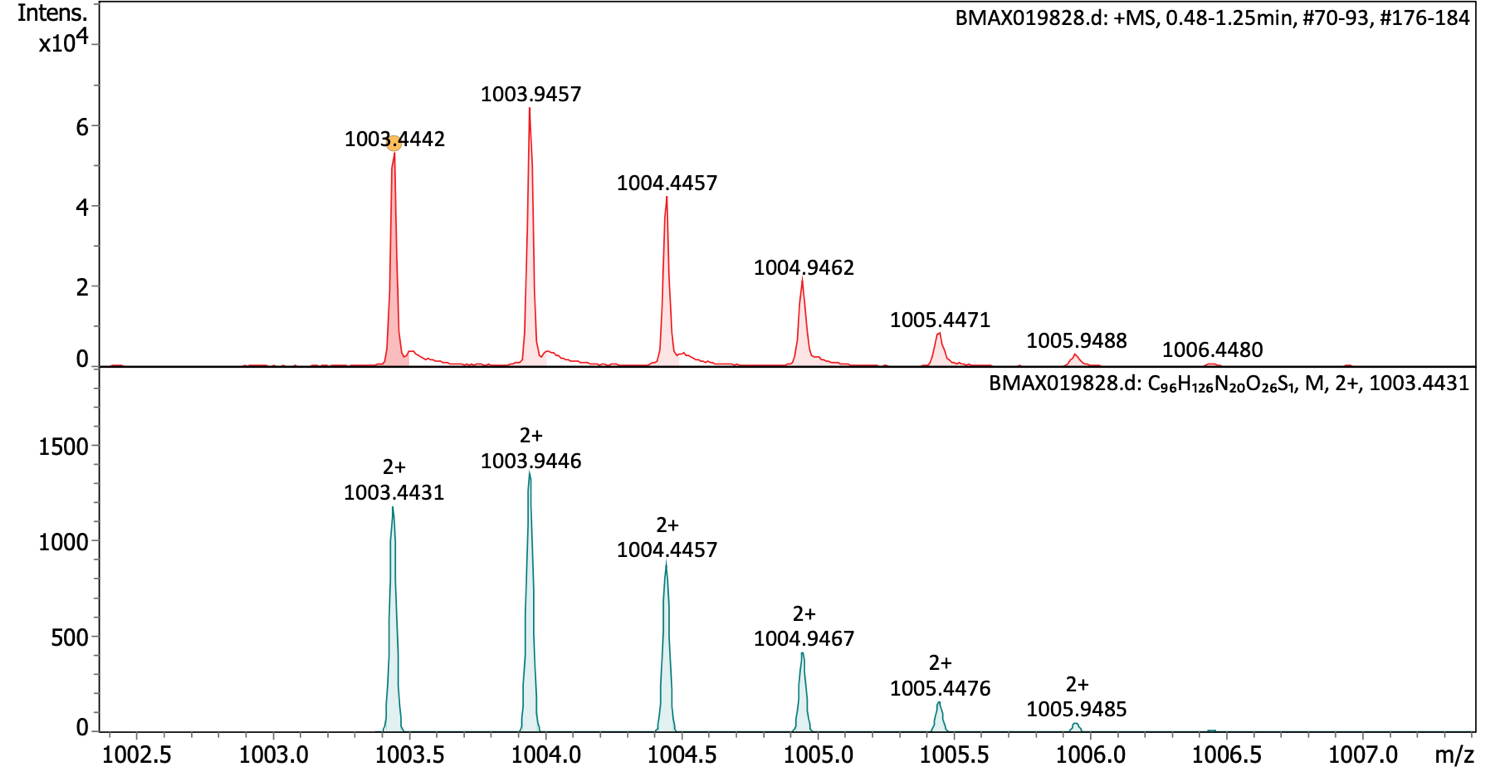


**Figure 14 Characterization of fluorescein-pep3–L–CONH_2_** HRMS (ESI) spectrum of purified peptide showing recorded mass spectrum (upper panel) and calculated spectrum (lower panel).

Synthesis of fluorescein-pep3–M–CONH_2_

**Fluorescein-KKYRYDVPDYSAM–CONH_2_**

The peptide was obtained as an orange solid and used without preparative RP-HPLC purification.

HRMS (ESI): calculated for [C_95_H_124_N_20_O_26_S_2_]^2+^: m/z 1012.4213, found: m/z 1012.4243


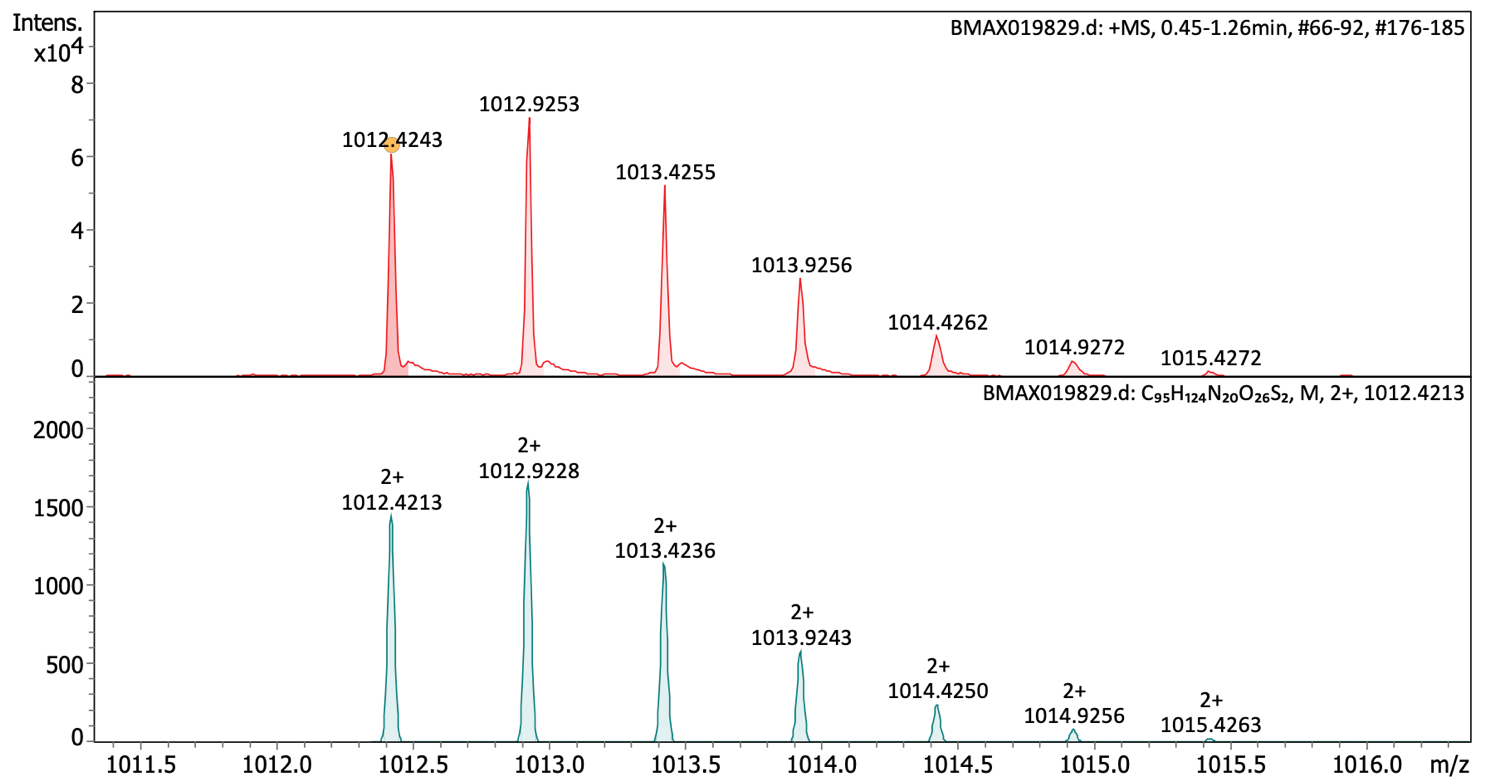


**Characterization of fluorescein-pep3–M–CONH_2_** HRMS (ESI) spectrum of purified peptide showing recorded mass spectrum (upper panel) and calculated spectrum (lower panel).

Synthesis of fluorescein-pep3–N–CONH_2_

**Fluorescein-KKYRYDVPDYSAN–CONH_2_**

The peptide was obtained as an orange solid and used without preparative RP-HPLC purification.

HRMS (ESI): calculated for [C_94_H_121_N_21_O_27_S_1_]^2+^: m/z 1003.9225, found: m/z 1003.9235


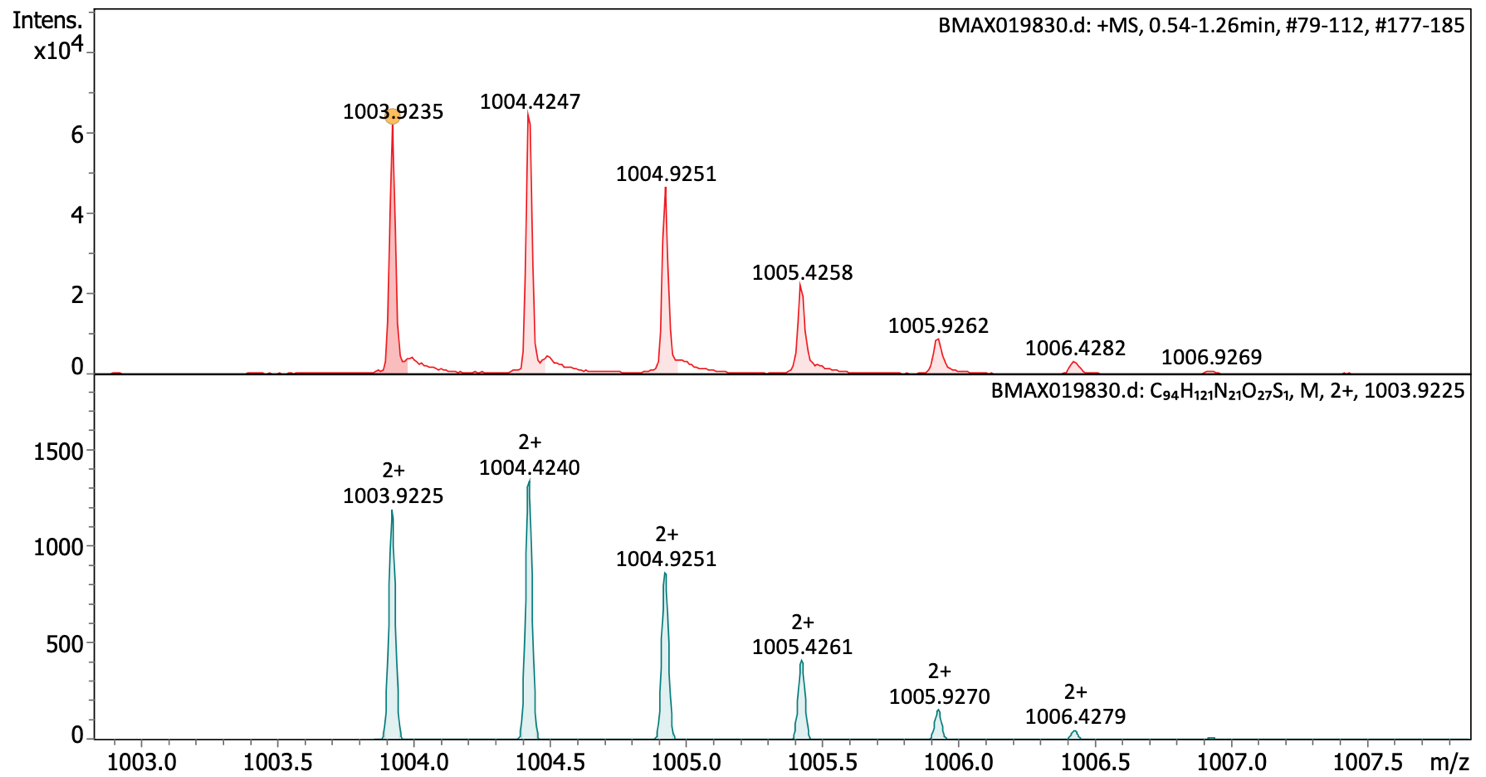


**Characterization of fluorescein-pep3–N–CONH_2_** HRMS (ESI) spectrum of purified peptide showing recorded mass spectrum (upper panel) and calculated spectrum (lower panel).

Synthesis of fluorescein-pep3–P–CONH_2_

**Fluorescein-KKYRYDVPDYSAP–CONH_2_**

The peptide was obtained as an orange solid and used without preparative RP-HPLC purification.

HRMS (ESI): calculated for [C_95_H_122_N_20_O_26_S_1_]^2+^: m/z 995.4274, found: m/z 995.4289


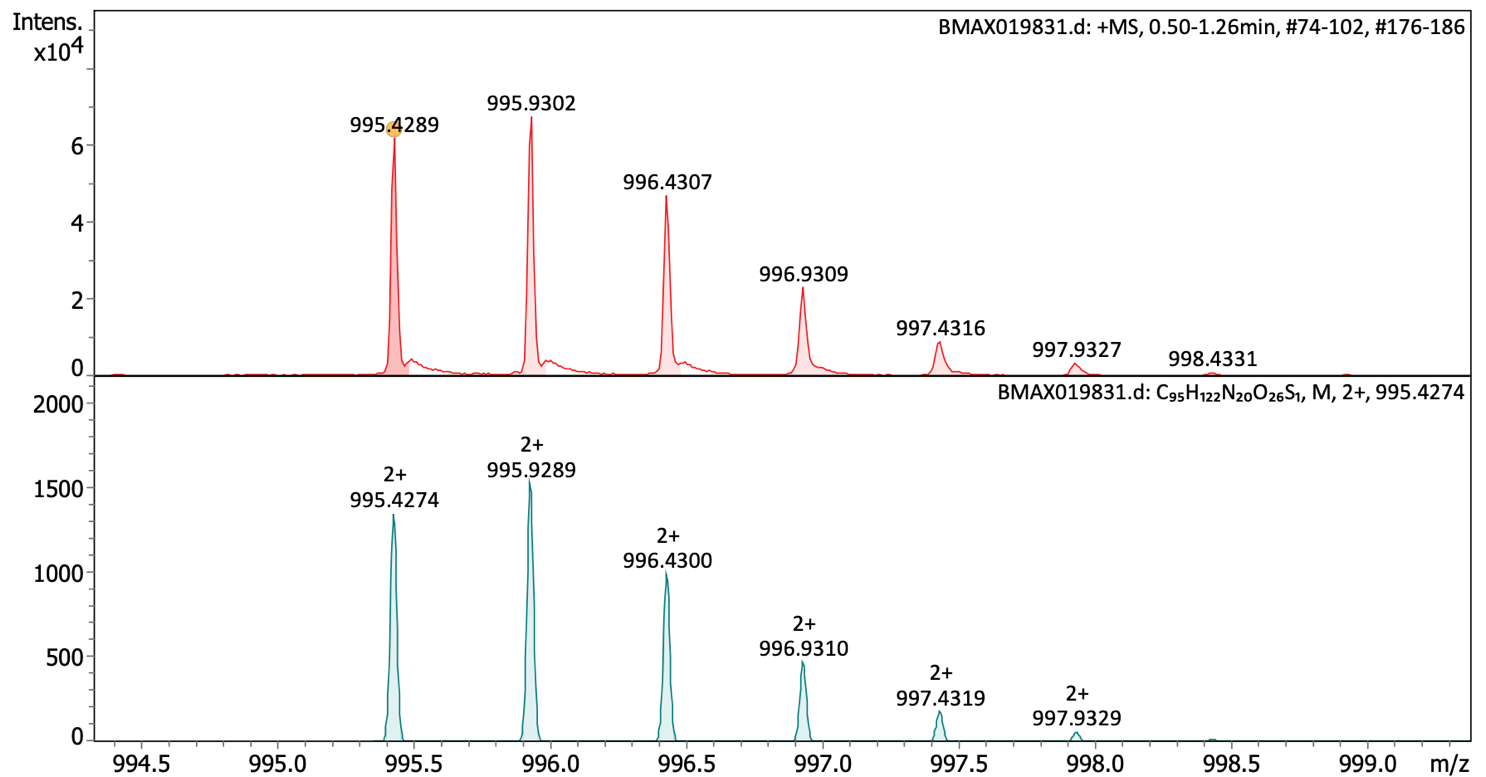


**Characterization of fluorescein-pep3–P–CONH_2_** HRMS (ESI) spectrum of purified peptide showing recorded mass spectrum (upper panel) and calculated spectrum (lower panel).

Synthesis of fluorescein-pep3–Q–CONH_2_

**Fluorescein-KKYRYDVPDYSAQ–CONH_2_**

The peptide was obtained as an orange solid and used without preparative RP-HPLC purification.

HRMS (ESI): calculated for [C_95_H_124_N_21_O_27_S_1_]2^+^: m/z 674.2893, found: m/z 674.2893

**
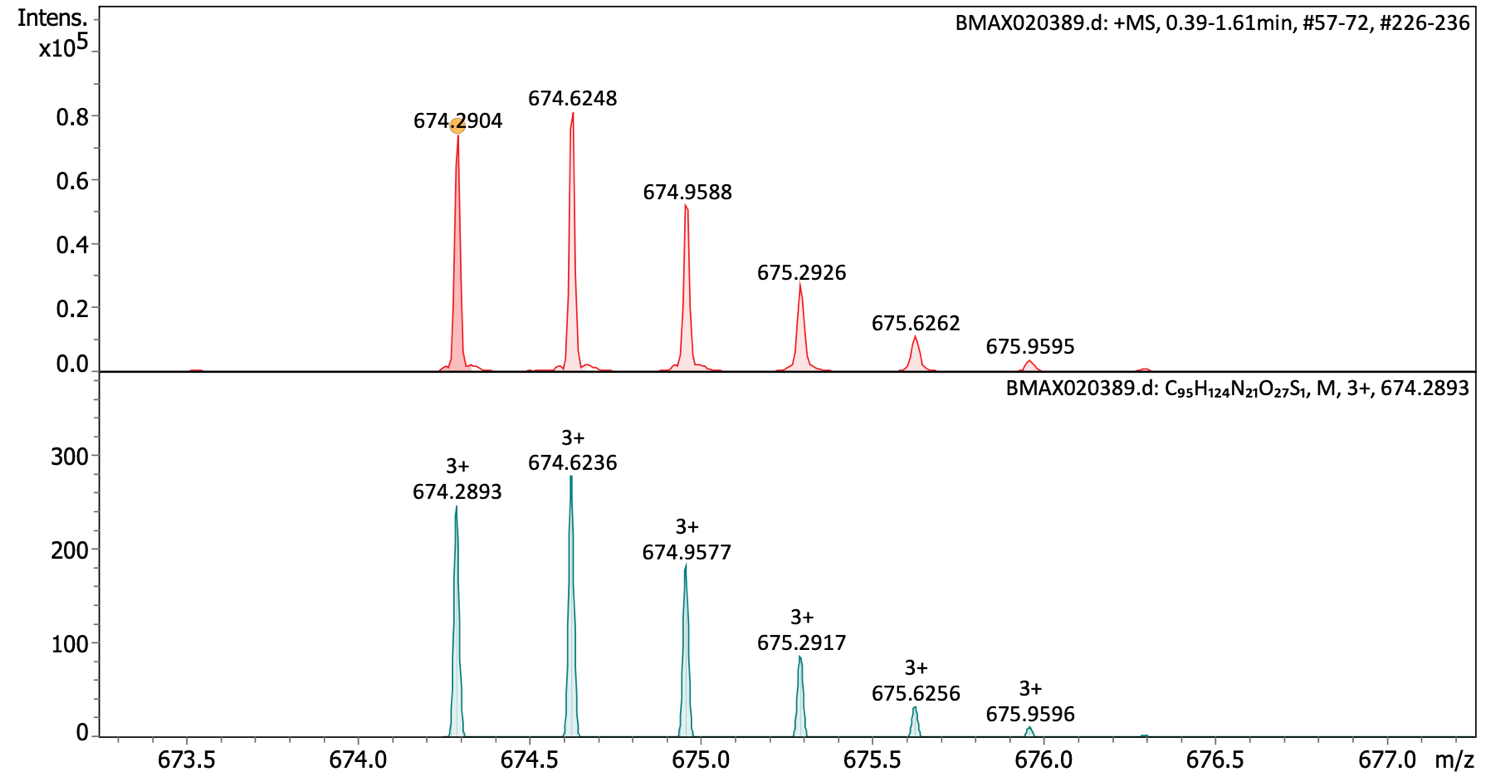
**

**Characterization of fluorescein-pep3–Q–CONH_2_** HRMS (ESI) spectrum of purified peptide showing recorded mass spectrum (upper panel) and calculated spectrum (lower panel).

Synthesis of fluorescein-pep3–R–CONH_2_

**Fluorescein-KKYRYDVPDYSAR–CONH_2_**

The peptide was obtained as an orange solid and used without preparative RP-HPLC purification.

HRMS (ESI): calculated for [C_96_H_127_N_23_O_26_S_1_]^2+^: m/z 1024.9516, found: m/z 1024.9523


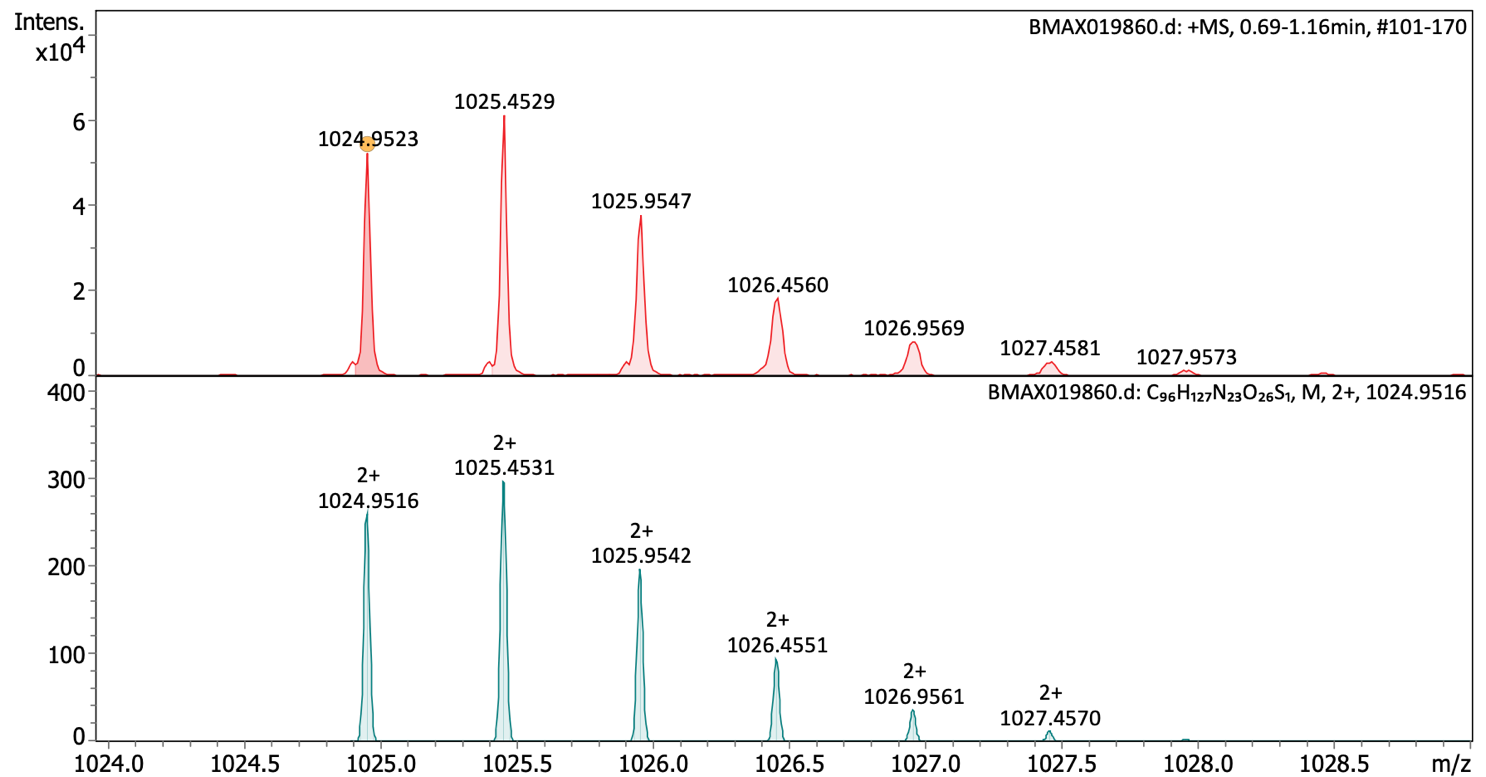


**Characterization of fluorescein-pep3–R–CONH_2_** HRMS (ESI) spectrum of purified peptide showing recorded mass spectrum (upper panel) and calculated spectrum (lower panel).

Synthesis of fluorescein-pep3–S–CONH_2_

**Fluorescein-KKYRYDVPDYSAS–CONH_2_**

The peptide was obtained as an orange solid and used without preparative RP-HPLC purification.

HRMS (ESI): calculated for [C_93_H_120_N_20_O_27_S_1_]^2+^: m/z 990.4171, found: m/z 990.4171


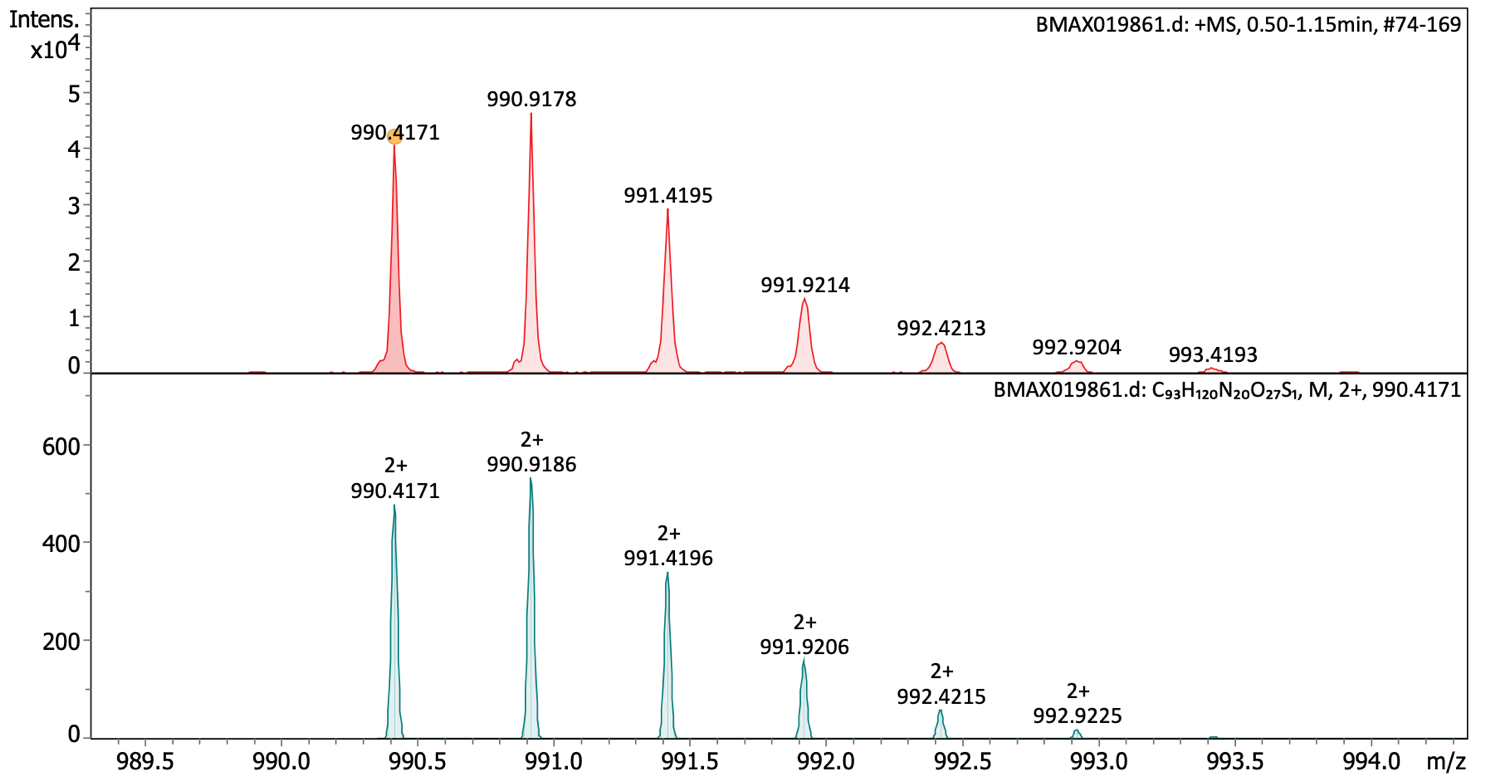


**Characterization of fluorescein-pep3–S–CONH_2_** HRMS (ESI) spectrum of purified peptide showing recorded mass spectrum (upper panel) and calculated spectrum (lower panel).

Synthesis of fluorescein-pep3–T–CONH_2_

**Fluorescein-KKYRYDVPDYSAT–CONH_2_**

The peptide was obtained as an orange solid and used without preparative RP-HPLC purification.

HRMS (ESI): calculated for [C_94_H_122_N_20_O_27_S_1_]2^+^: m/z 997.4249, found: m/z 997.4243


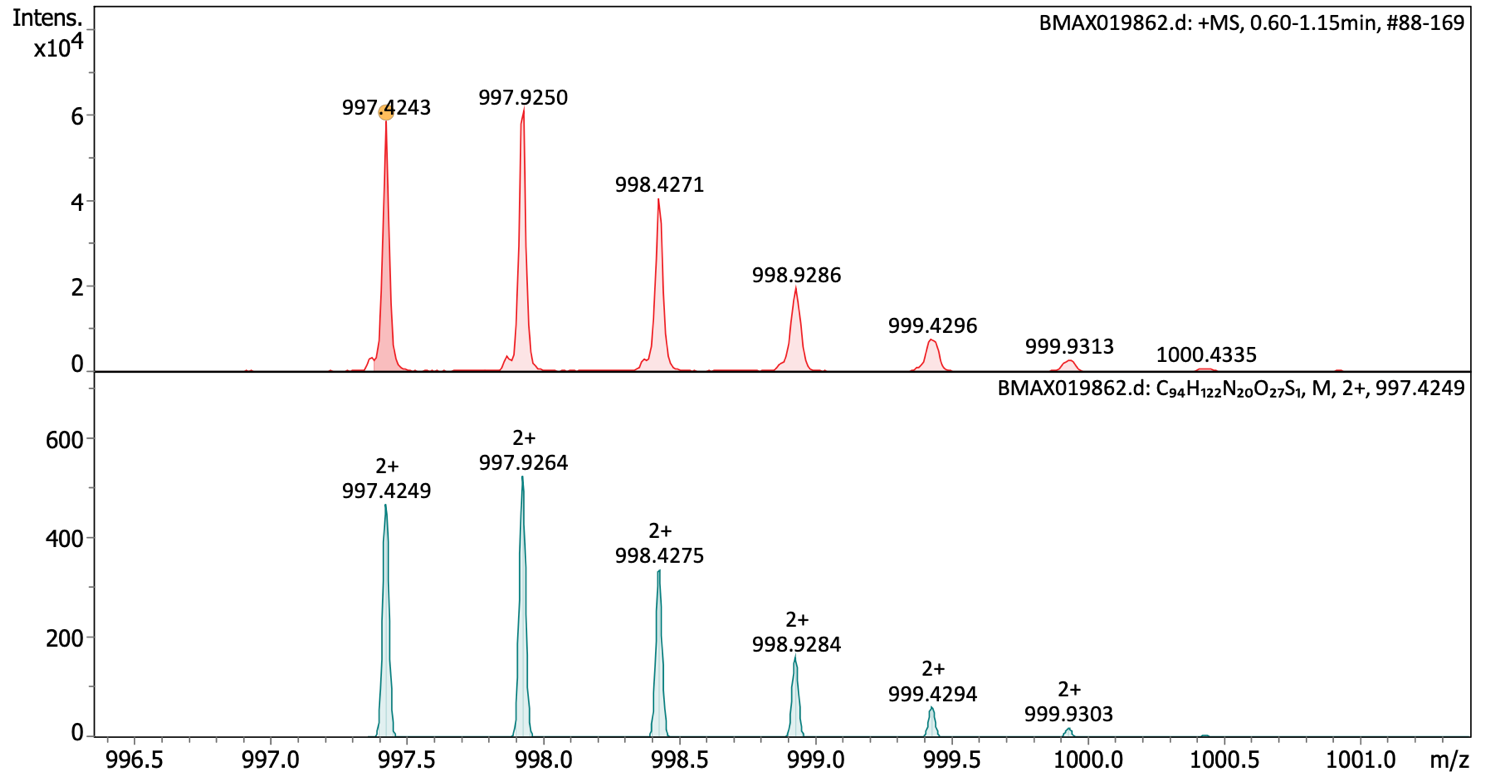


**Characterization of fluorescein-pep3–T–CONH_2_** HRMS (ESI) spectrum of purified peptide showing recorded mass spectrum (upper panel) and calculated spectrum (lower panel).

Synthesis of fluorescein-pep3–V–CONH_2_

**Fluorescein-KKYRYDVPDYSAV–CONH_2_**

The peptide was obtained as an orange solid and used without preparative RP-HPLC purification.

HRMS (ESI): calculated for [C_95_H_124_N_20_O_26_S_1_]^2+^: m/z 996.4353, found: m/z 996.4367


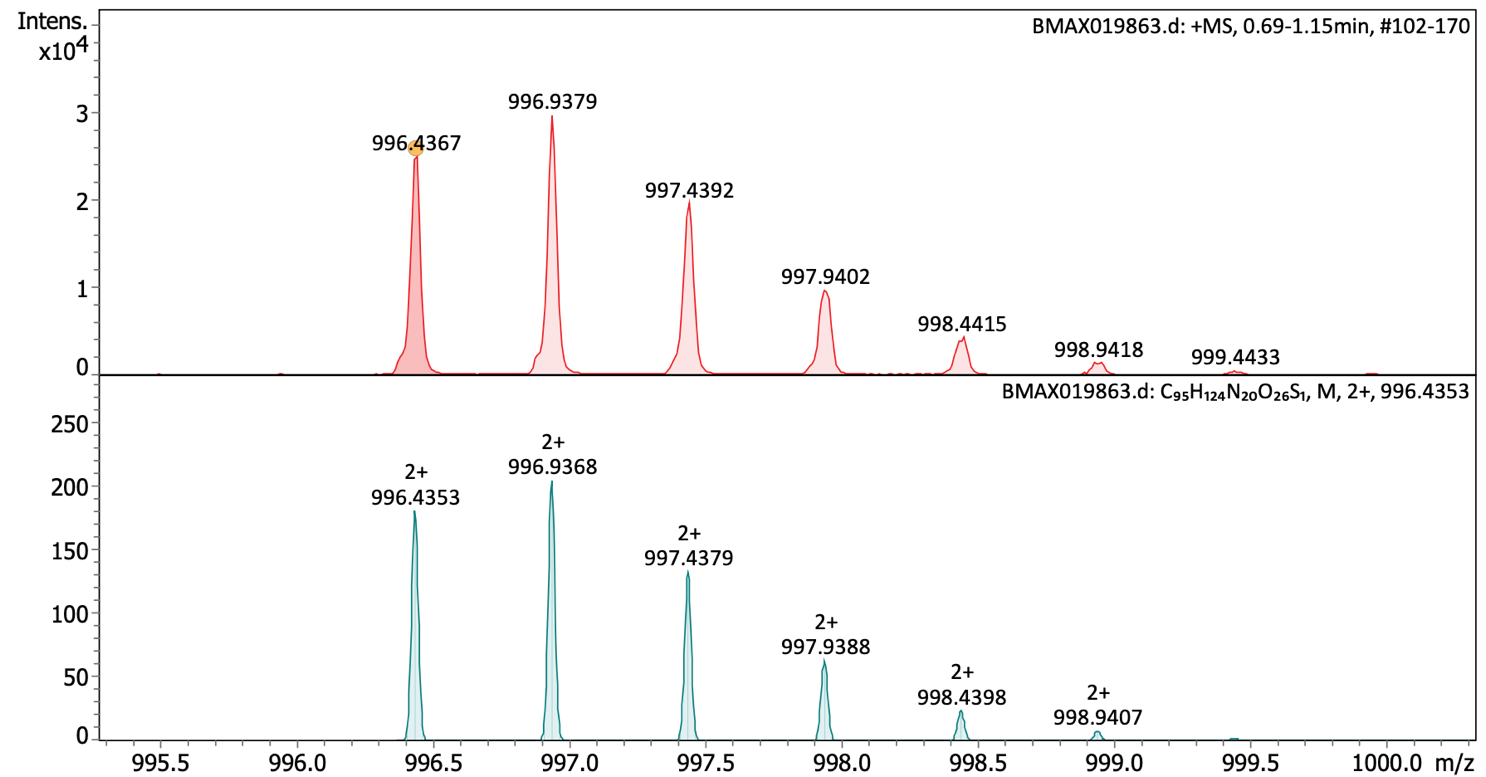


**Characterization of fluorescein-pep3–V–CONH_2_** HRMS (ESI) spectrum of purified peptide showing recorded mass spectrum (upper panel) and calculated spectrum (lower panel).

Synthesis of fluorescein-pep3–W–CONH_2_

**Fluorescein-KKYRYDVPDYSAW–CONH_2_**

The peptide was obtained as an orange solid and used without preparative RP-HPLC purification.

HRMS (ESI): calculated for [C_101_H_125_N_21_O_26_S_1_]^2+^: m/z 1039.9407, found: m/z 1039.9385


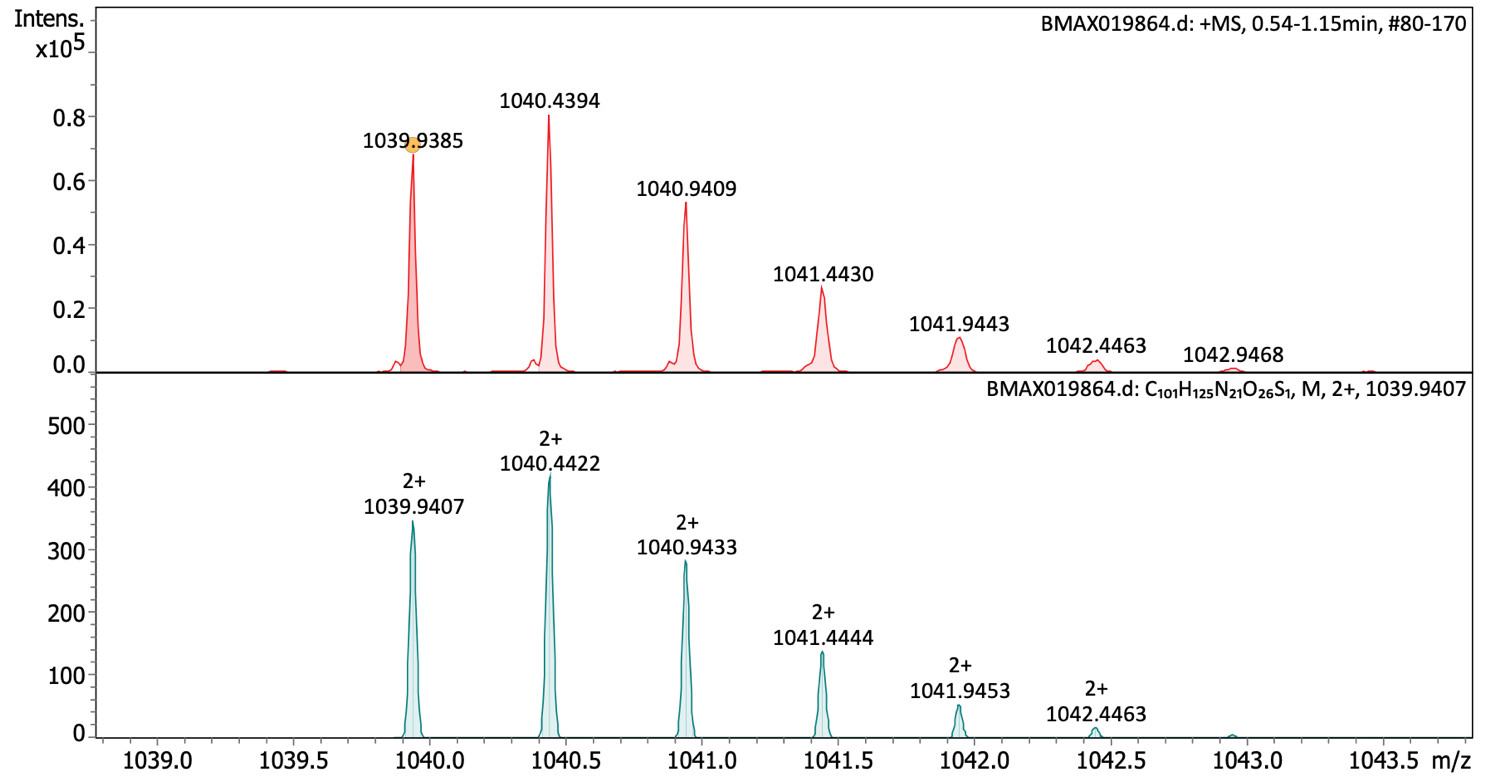


**Characterization of fluorescein-pep3–W–CONH_2_** HRMS (ESI) spectrum of purified peptide showing recorded mass spectrum (upper panel) and calculated spectrum (lower panel).

Synthesis of fluorescein-pep3–Y–CONH_2_

**Fluorescein-KKYRYDVPDYSAY–CONH_2_**

The peptide was obtained as an orange solid and used without preparative RP-HPLC purification.

HRMS (ESI): calculated for [C_99_H_124_N_20_O_27_S_1_]^2+^: m/z 1028.4327, found: m/z 1028.4341


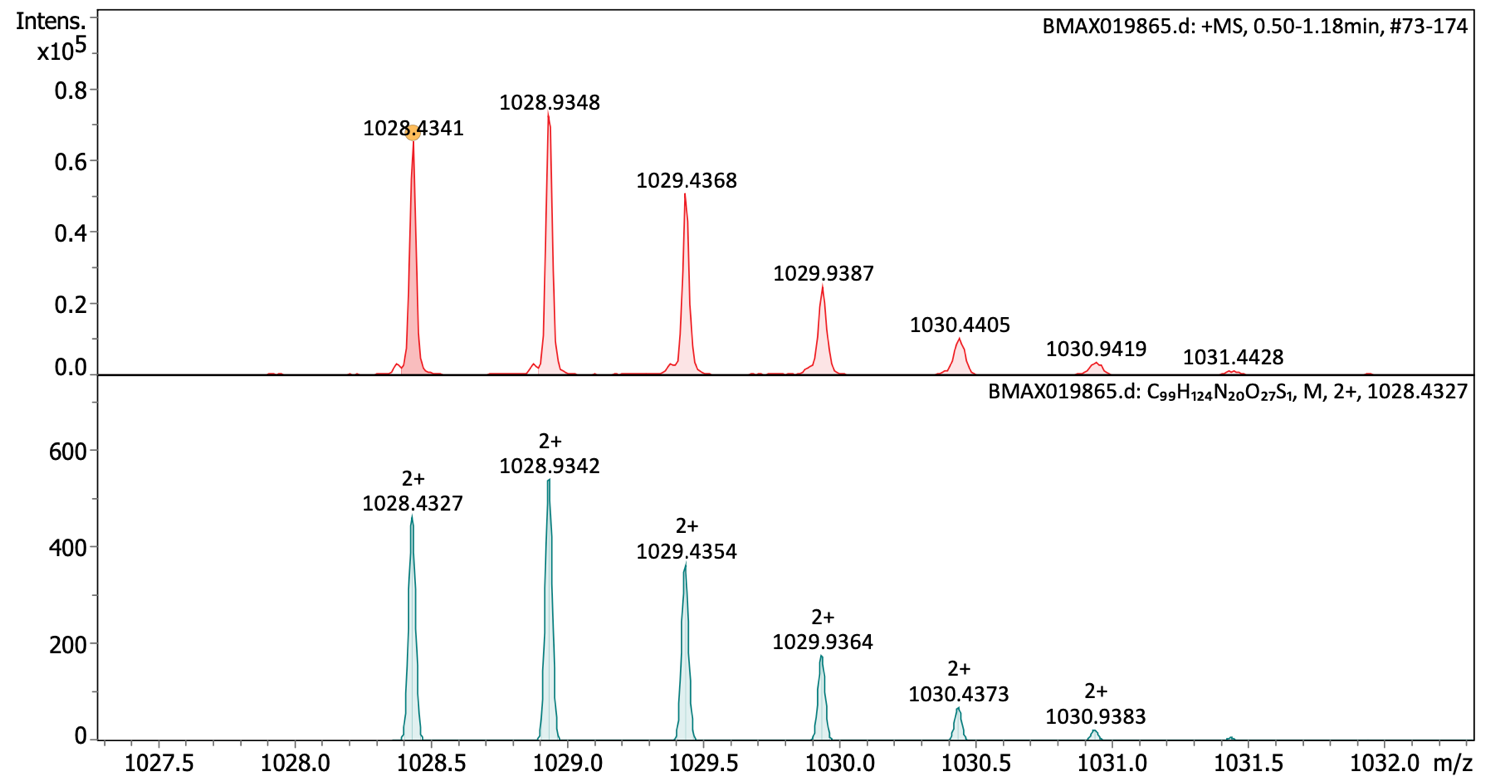


**Characterization of fluorescein-pep3–Y–CONH_2_** HRMS (ESI) spectrum of purified peptide showing recorded mass spectrum (upper panel) and calculated spectrum (lower panel).

Synthesis of fluorescein-pep3–N–COOH

**Fluorescein-KKYRYDVPDYSAN–COOH**

The peptide was obtained as an orange solid.

HRMS (ESI): calculated for [C_94_H_120_N_20_O_28_S_1_]^2+^: m/z 1004.4145, found: m/z 1004.4155

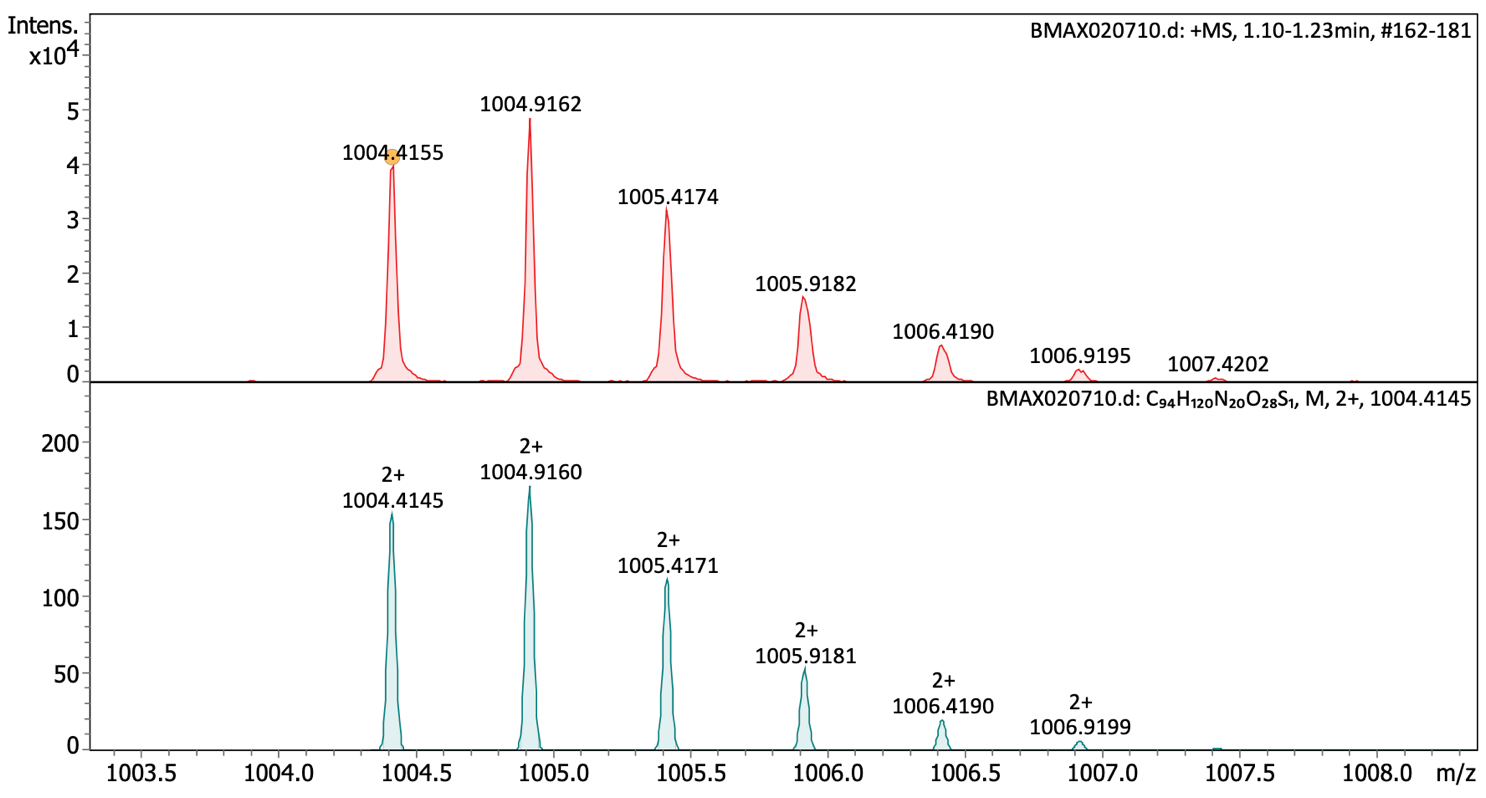


**Characterization of fluorescein-pep3–N–COOH** Analytical RP-HPLC of purified peptide. HRMS (ESI) spectrum of purified peptide showing recorded mass spectrum (upper panel) and calculated spectrum (lower panel).

Synthesis of fluorescein-pep3–Q–COOH

**Fluorescein-KKYRYDVPDYSAQ–COOH**

The peptide was obtained as an orange solid.

HRMS (ESI): calculated for [C_95_H_122_N_20_O_28_S_1_]^2+^: m/z 1011.4224, found: m/z 1011.4222

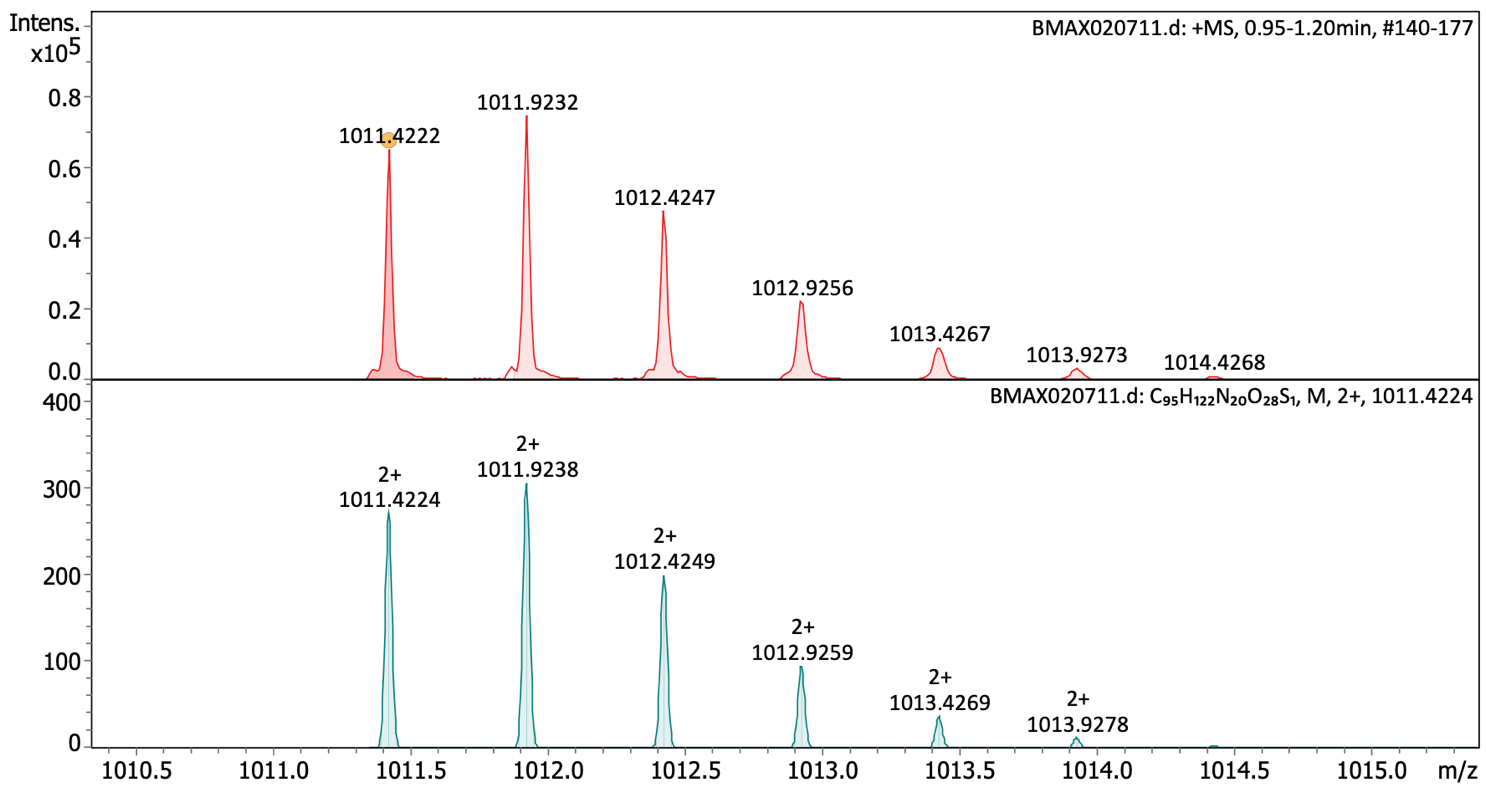


**Characterization of fluorescein-pep3–Q–COOH** Analytical RP-HPLC of purified peptide. HRMS (ESI) spectrum of purified peptide showing recorded mass spectrum (upper panel) and calculated spectrum (lower panel).

Synthesis of fluorescein-pep3–R–CH_2_OH

**Fluorescein-** **KEEDEKGSRASDDFRDLR–CH_2_OH**

The peptide was obtained as an orange solid.

HRMS (ESI): calculated for [C_108_H_157_N_30_O_39_S_1_]^3+^: m/z 843.3643, found: m/z 843.3649

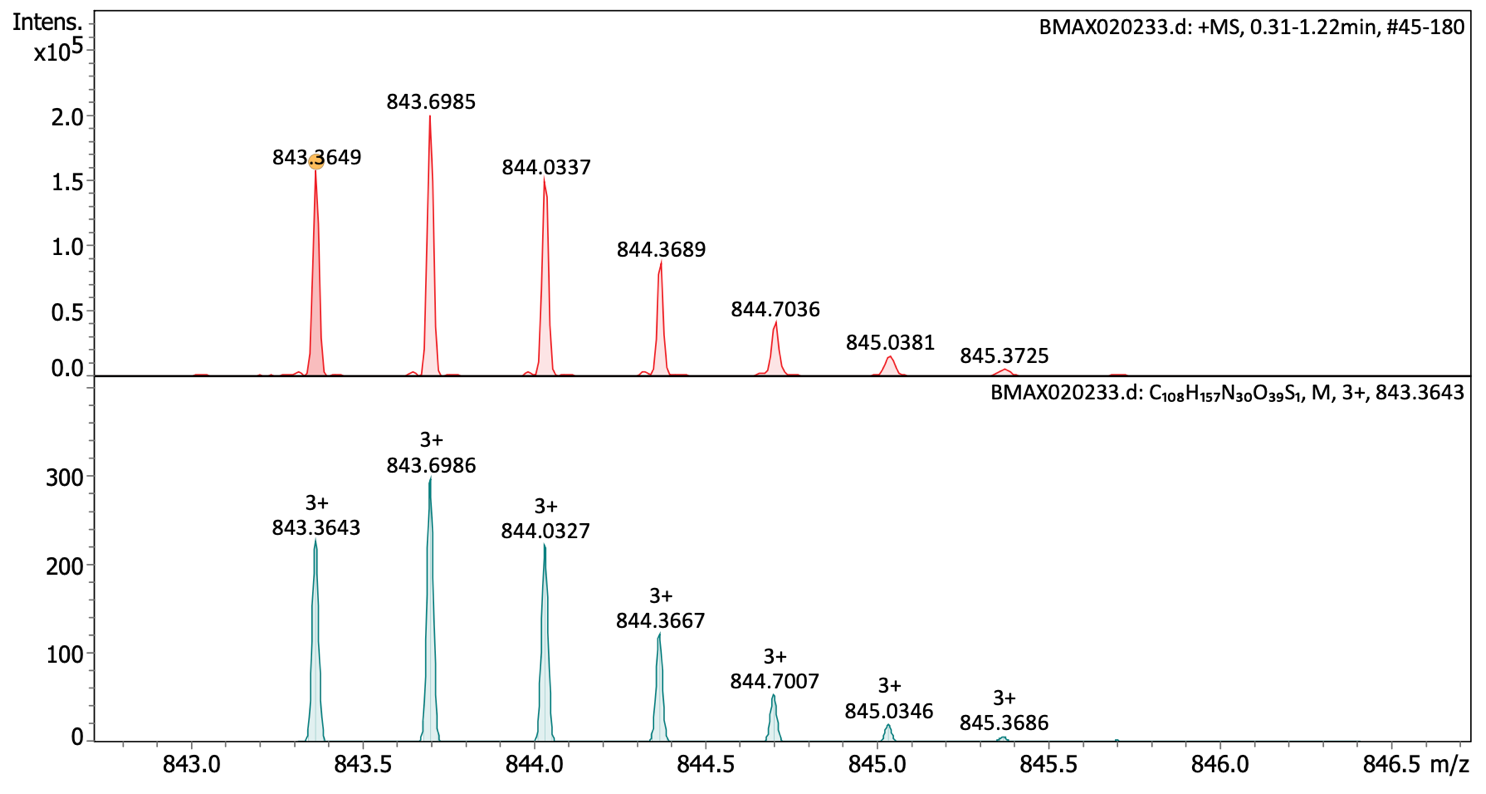


**Characterization of fluorescein-pep3–R–CH_2_OH** Analytical RP-HPLC of purified peptide. HRMS (ESI) spectrum of purified peptide showing recorded mass spectrum (upper panel) and calculated spectrum (lower panel).

Synthesis of fluorescein-pep3–R–COOMe

**Fluorescein-** **KEEDEKGSRASDDFRDLR–COOMe**

The peptide was obtained as an orange solid.

HRMS (ESI): calculated for [C_109_H_155_N_30_O_40_S_1_]^3+^: m/z 852.0240, found: m/z 852.0253

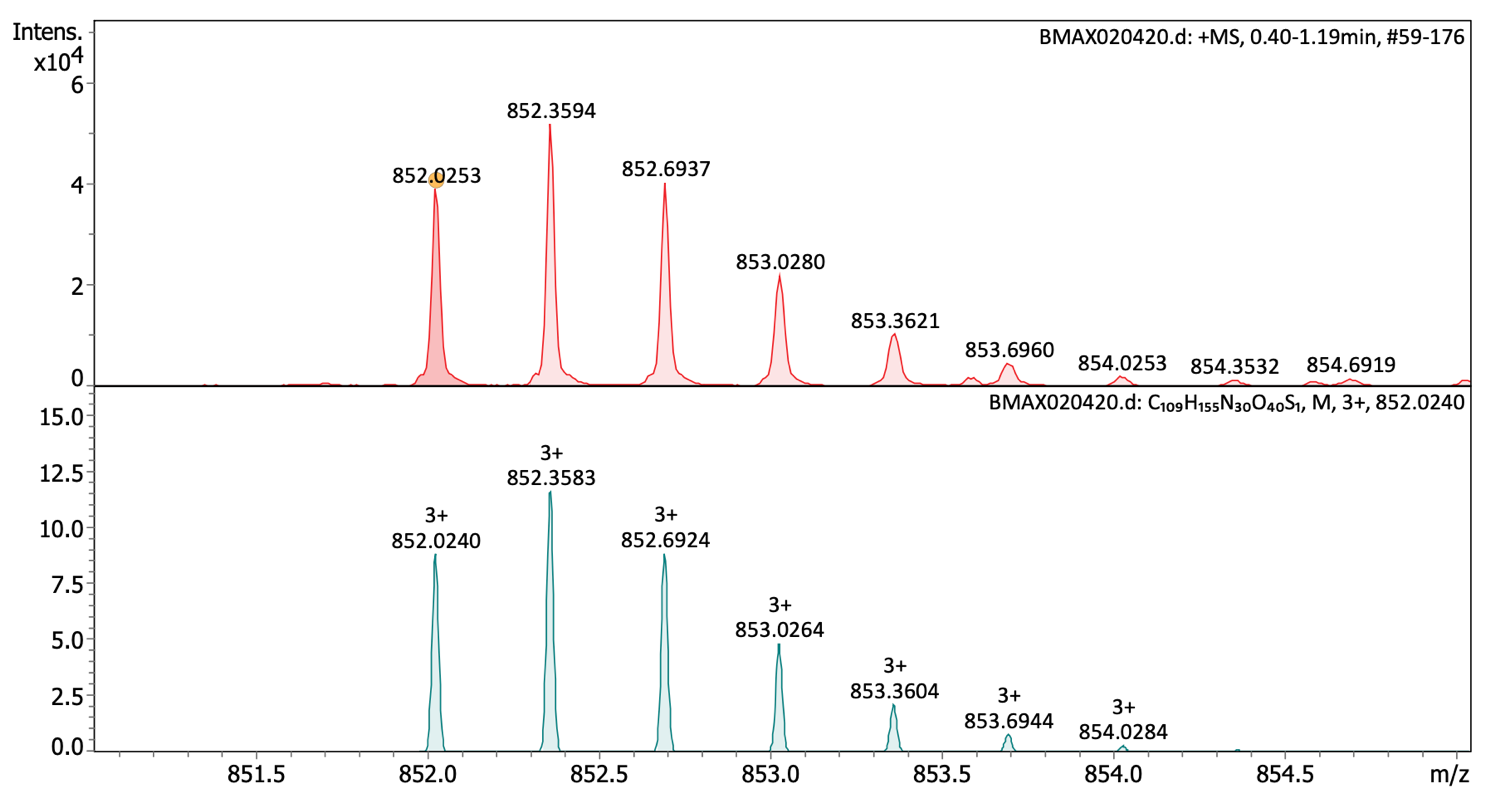


**Characterization of fluorescein-pep3–R–COOMe** Analytical RP-HPLC of purified peptide. HRMS (ESI) spectrum of purified peptide showing recorded mass spectrum (upper panel) and calculated spectrum (lower panel).

### Characterization of SPPS products for sortase reaction

Synthesis of pep1 [homocitrulline (HCT)]–D–COOH

**GGGKDLEGKGGS[HCT]GSGS[HCT]GGSKYPYDVPDYAKD–COOH**

The peptide was obtained as a white solid.

HRMS (ESI): calculated for [C_142_H_219_N_41_O_53_]^+^: 3346.5702, found: m/z 3346.5837

**
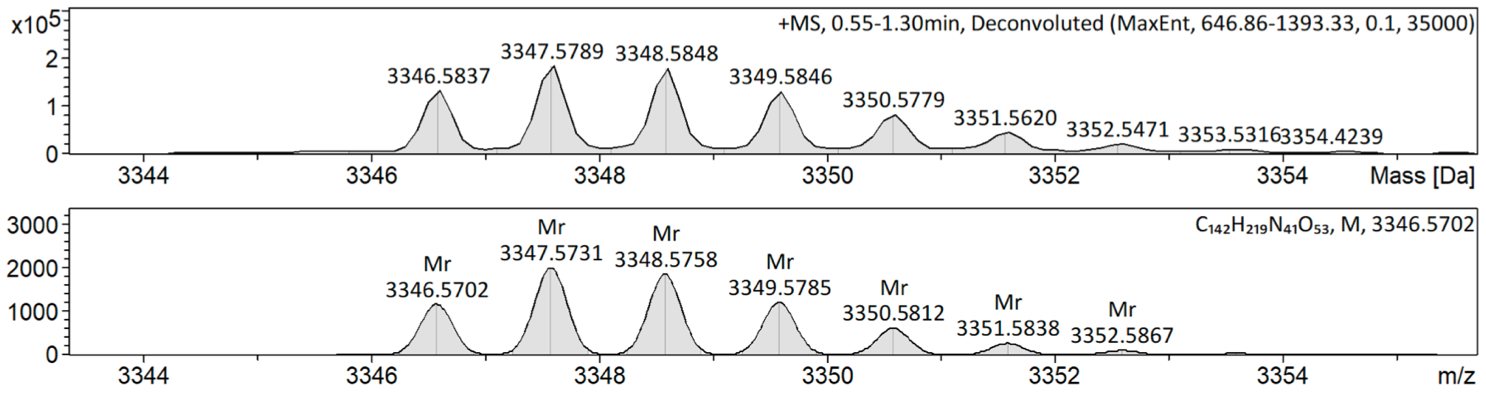
**

**Characterization of pep1 [HCT]–D–COOH** Analytical RP-HPLC of purified peptide. HRMS (ESI) spectrum of purified peptide showing recorded mass spectrum (upper panel) and calculated spectrum (lower panel).

Synthesis of pep1 [L-DOP (LDO)]–D–COOH

**GGGKDLEGKGGS[LDO]GSGS[LDO]GGSKYPYDVPDYAKD–COOH**

The peptide was obtained as a white solid.

HRMS (ESI): calculated for [C_146_H_211_N_37_O_55_]^+^: 3362.4851, found: m/z 3362.4961

**
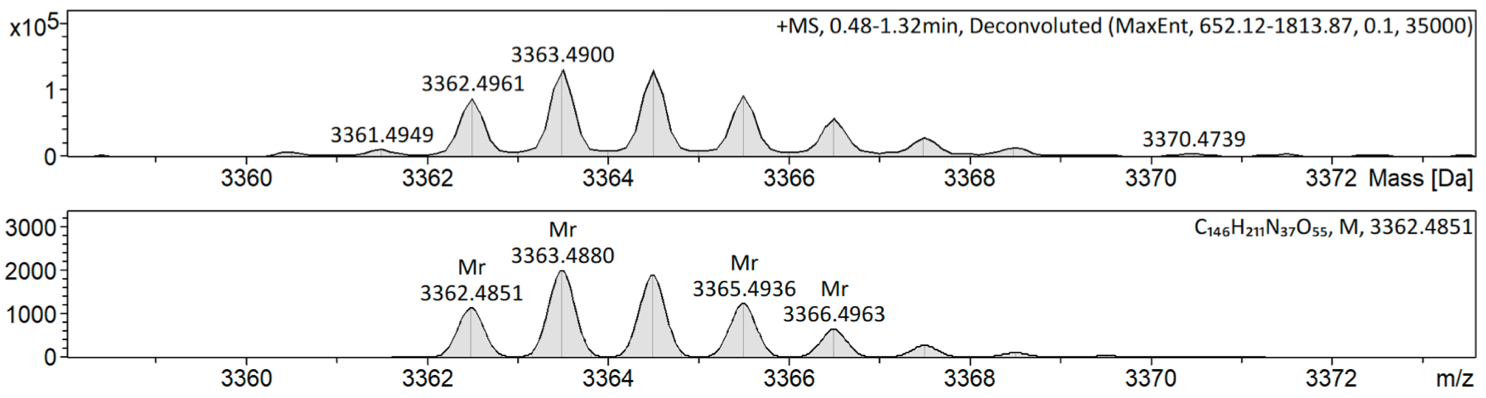
**

**Characterization of pep1 [LDO]–D–COOH** Analytical RP-HPLC of purified peptide. HRMS (ESI) spectrum of purified peptide showing recorded mass spectrum (upper panel) and calculated spectrum (lower panel).

Synthesis of pep1 [carboxymethyllysine (CML)]–D–COOH

**GGGKDLEGKGGS[CML]GSGS[CML]GGSKYPYDVPDYAKD–COOH**

The peptide was obtained as a white solid.

HRMS (ESI): calculated for [C_144_H_221_N_39_O_55_]^+^: m/z 3376.5695, found: m/z 3376.5792

**
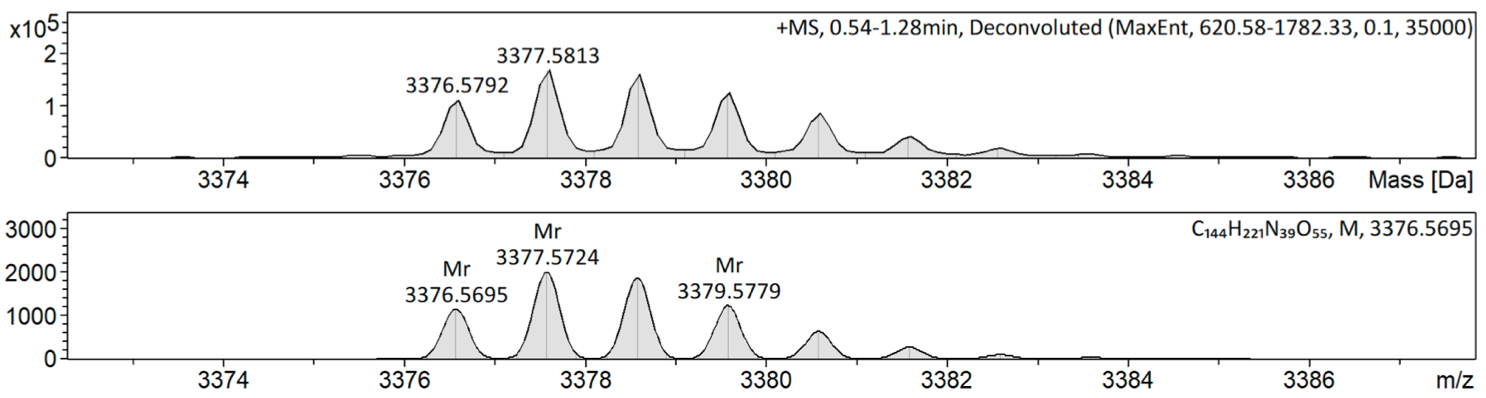
**

**Characterization of pep1 [CML]–D–COOH** Analytical RP-HPLC of purified peptide. HRMS (ESI) spectrum of purified peptide showing recorded mass spectrum (upper panel) and calculated spectrum (lower panel).

Synthesis of pep1 [A]–S–COOH

**GGGKDLEGKGGSAGSGSAGGSKYPYDVPDYAKS-COOH**

The peptide was obtained as a white solid.

HRMS (ESI): calculated for [C_133_H_203_N_37_O_50_]^+^: m/z 3118.4480 found: m/z 3118.4579

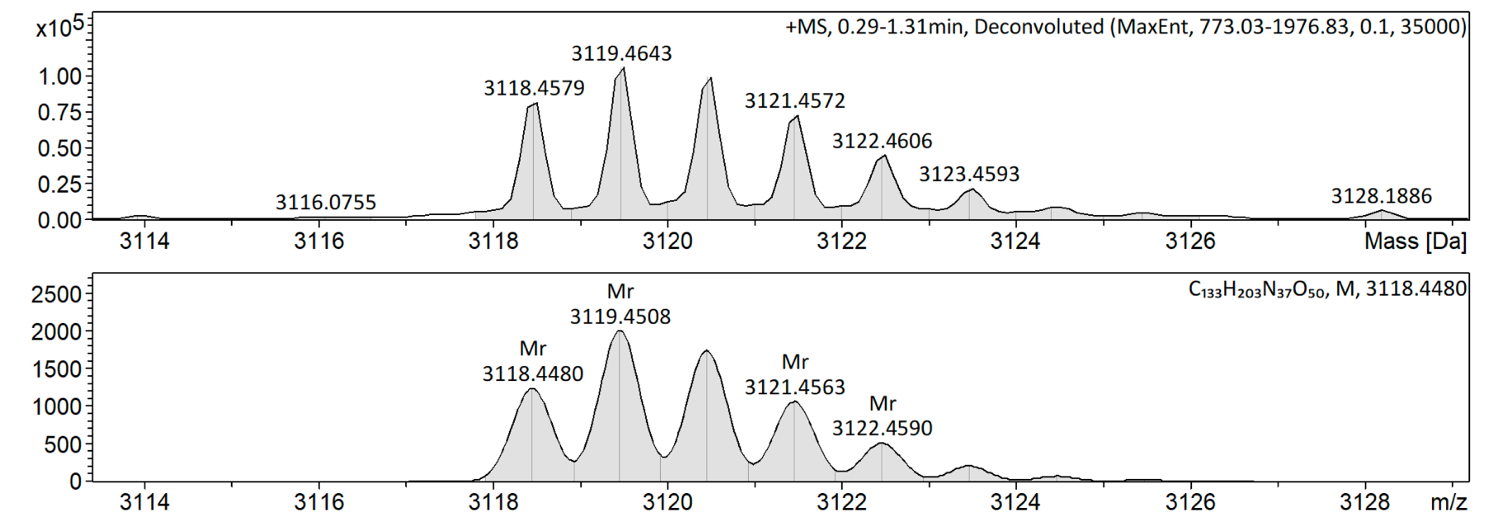


**Characterization of pep1–S–COOH** Analytical RP-HPLC of purified peptide. HRMS (ESI) spectrum of purified peptide showing recorded mass spectrum (upper panel) and calculated spectrum (lower panel).

Synthesis of pep1 [A]–S–CONH_2_

**GGGKDLEGKGGSAGSGSAGGSKYPYDVPDYAKS-CONH_2_**

The peptide was obtained as a white solid.

HRMS (ESI): calculated for [C_133_H_204_N_38_O_49_]^+^: m/z 3117.4639 found: m/z 3117.4830

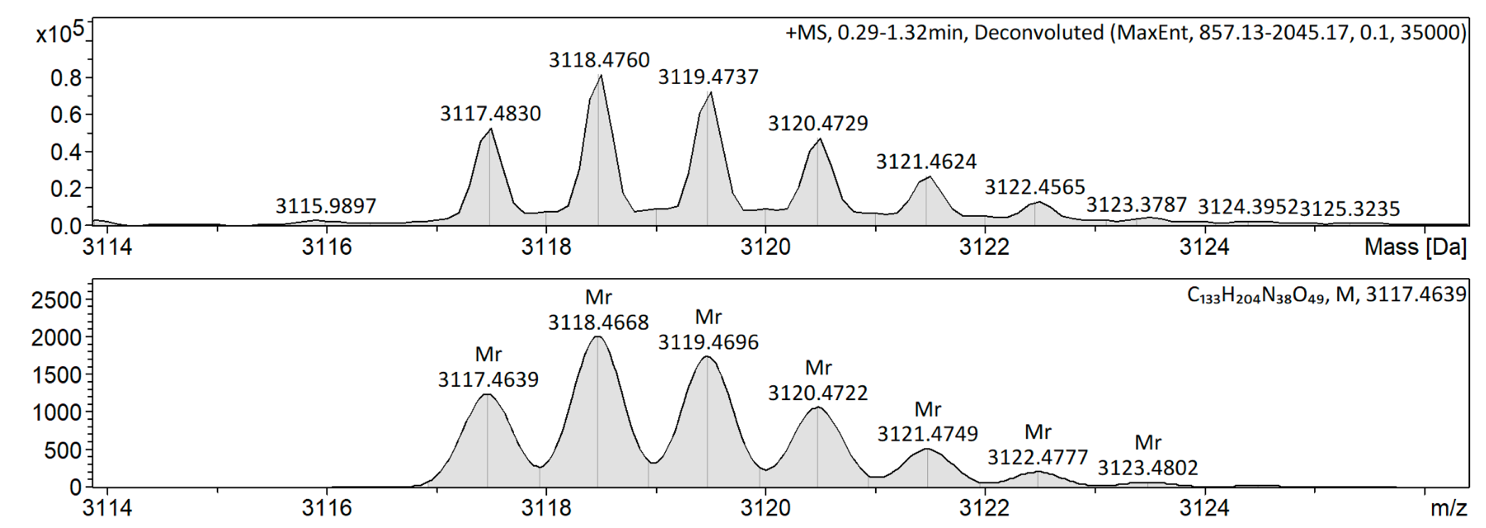


**Characterization of pep1–S–CONH_2_** Analytical RP-HPLC of purified peptide. HRMS (ESI) spectrum of purified peptide showing recorded mass spectrum (upper panel) and calculated spectrum (lower panel).

Synthesis of pep1 [A]–D–COOH

**GGGKDLEGKGGSAGSGSAGGSKYPYDVPDYAKD–COOH**

The peptide was obtained as a white solid.

HRMS (ESI): calculated for [C_134_H_203_N_37_O_51_]^+^: m/z 3146.4429, found: m/z 3146.4586

**
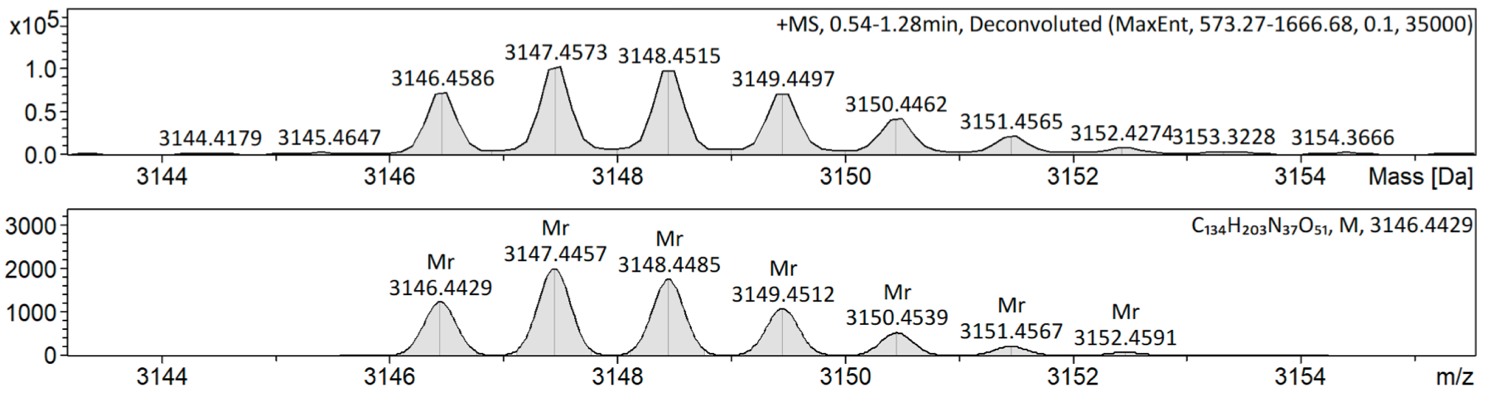
**

**Characterization of pep1 [A]–D–COOH** Analytical RP-HPLC of purified peptide. HRMS (ESI) spectrum of purified peptide showing recorded mass spectrum (upper panel) and calculated spectrum (lower panel).

Synthesis of pep1 [hexanoyllysine (KHL)]–D–COOH

**GGGKDLEGKGGS[KHL]GSGS[KHL]GGSKYPYDVPDYAKD–COOH**

The peptide was obtained as a white solid.

HRMS (ESI): calculated for [C_152_H_237_N_39_O_53_]^+^: m/z 3456.7049, found: m/z 3456.7200

**
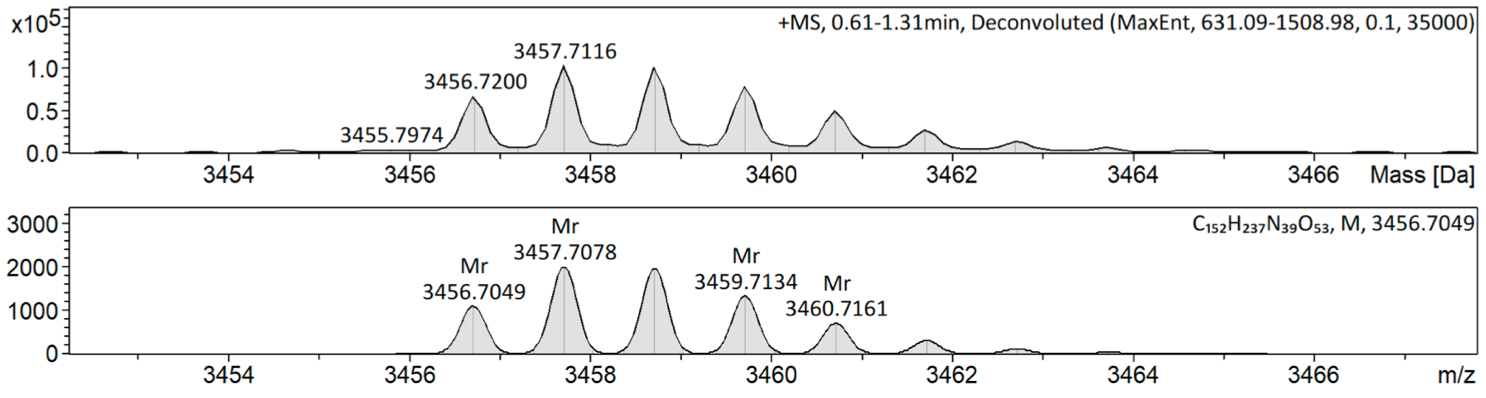
**

**Characterization of pep1 [KHL]–D–COOH** Analytical RP-HPLC of purified peptide. HRMS (ESI) spectrum of purified peptide showing recorded mass spectrum (upper panel) and calculated spectrum (lower panel).

Synthesis of pep2 [DxxD]–R–COOH

**GGGRRLEGKEEDEKGSRASDDFRDLR–COOH**

The peptide was obtained as a white solid.

HRMS (ESI): calculated for [C_118_H_197_N_43_O_45_]^2+^: m/z 1468.2219, found: m/z 1468.226

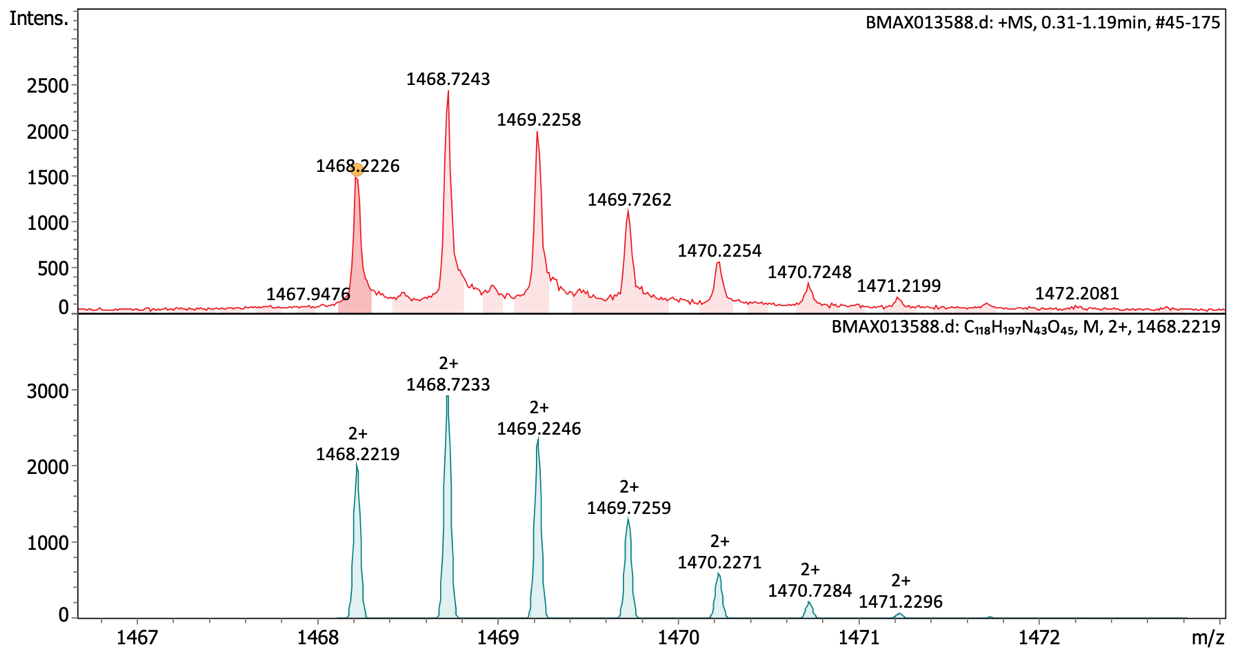


**Characterization of pep2 [DxxD]–R–COOH** Analytical RP-HPLC of purified peptide. HRMS (ESI) spectrum of purified peptide showing recorded mass spectrum (upper panel) and calculated spectrum (lower panel).

Synthesis of pep2 [DxxD]–R–CONH_2_

**GGGRRLEGKEEDEKGSRASDDFRDLR–CONH_2_**

The peptide was obtained as a white solid.

HRMS (ESI): calculated for [C_118_H_199_N_44_O_44_]^2+^: m/z 1467.7299, found: m/z 1467.7301

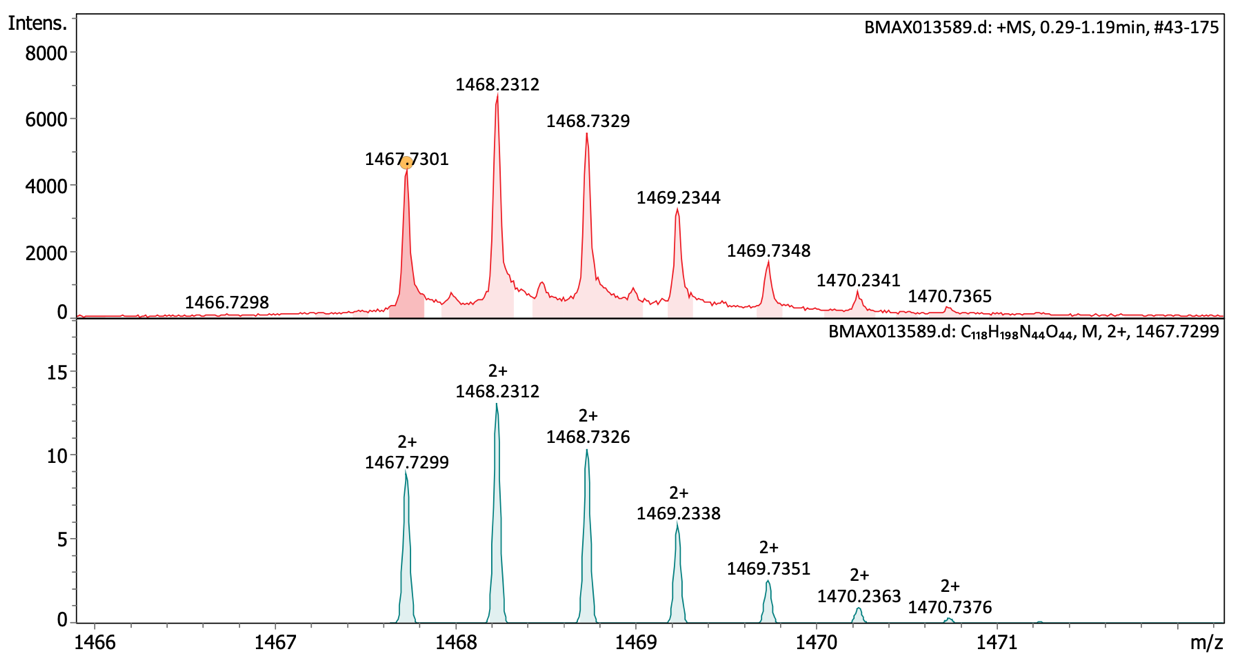


**Characterization of pep2 [DxxD]–R–CONH2** Analytical RP-HPLC of purified peptide. HRMS (ESI) spectrum of purified peptide showing recorded mass spectrum (upper panel) and calculated spectrum (lower panel).

Synthesis of pep2 [RxxG]–R–COOH

**GGGRRLEGKEEDEKGSRASDRFRGLR–COOH**

The peptide was obtained as a white solid.

HRMS (ESI): calculated for [C_118_H_200_N_46_O_41_]^+^: m/z 2917.4979, found: m/z 2917.5090

**
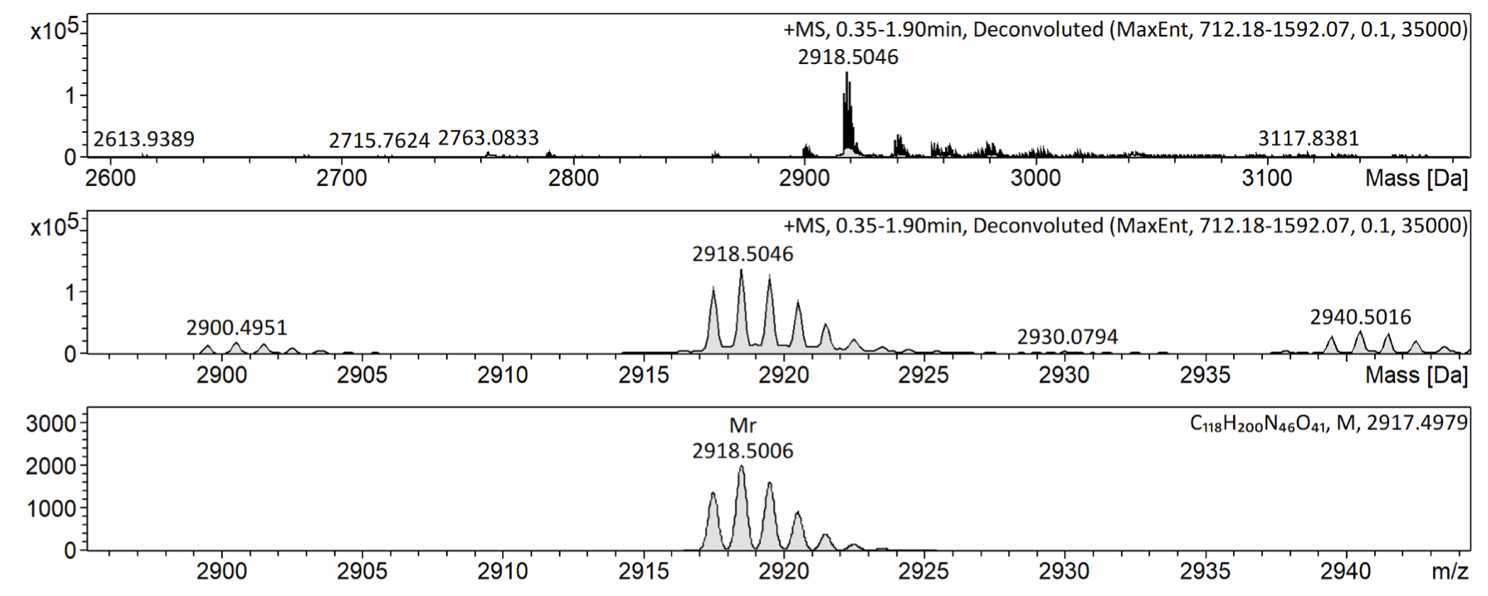
**

**Characterization of pep2 [RxxG]–R–COOH** Analytical RP-HPLC of purified peptide. HRMS (ESI) spectrum of purified peptide showing recorded mass spectrum (upper panel) and calculated spectrum (lower panel).

Synthesis of pep2 [RxxG]–R–CONH_2_

**GGGRRLEGKEEDEKGSRASDRFRGLR–CONH_2_**

The peptide was obtained as a white solid.

HRMS (ESI): calculated for [C_118_H_201_N_47_O_40_]^+^: m/z 2916.5139, found: m/z 2916.5309

**
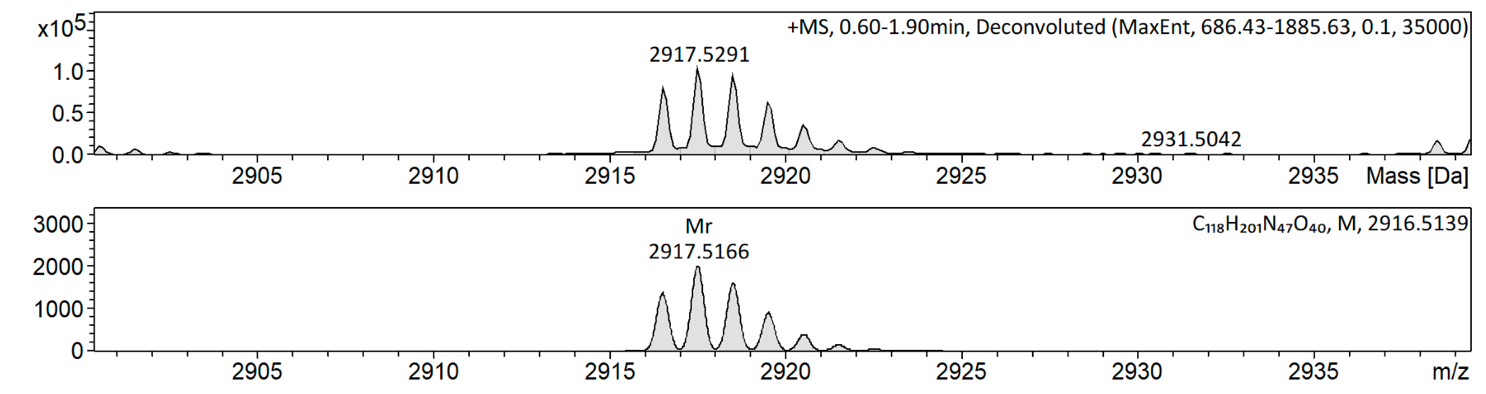
**

**Characterization of pep2 [RxxG]–R–CONH_2_** Analytical RP-HPLC of purified peptide. HRMS (ESI) spectrum of purified peptide showing recorded mass spectrum (upper panel) and calculated spectrum (lower panel).

### Characterization of protein-peptide conjugates

Preparation of GFP–GGGKDLEGKGGS[HCT]GSGS[HCT]GGSKYPYDVPDYAKD–COOH

Protein was prepared as described in the general method.

**ESI-MS characterization of protein conjugate sfGFP–pep1 [HCT]** Complex mass spectrum (left panel). Deconvoluted mass spectrum (right panel). Found: 30806.5, calculated for [C_1366_H_2113_N_371_O_428_S_7_]: 30805.0.

Preparation of GFP– GGGKDLEGKGGS[LDO]GSGS[LDO]GGSKYPYDVPDYAKD–COOH

Protein was prepared as described in the general method.

**ESI-MS characterization of protein conjugate sfGFP–pep1 [LDO]** Complex mass spectrum (left panel). Deconvoluted mass spectrum (right panel). Found: 30822.5, calculated for [C_1370_H_2105_N_369_O_430_S_7_]: 30820.9.

Preparation of GFP– GGGKDLEGKGGS[CML]GSGS[CML]GGSKYPYDVPDYAKD–COOH

Protein was prepared as described in the general method.

**ESI-MS characterization of protein conjugate sfGFP–pep1 [CML]** Complex mass spectrum (left panel). Deconvoluted mass spectrum (right panel). Found: 30836.0, calculated for [C_1368_H_2115_N_369_O_430_S_7_]: 30835.0.

Preparation of GFP– GGGKDLEGKGGSAGSGSAGGSKYPYDVPDYAKD–COOH

Protein was prepared as described in the general method.

**ESI-MS characterization of protein conjugate sfGFP–pep1 [A]–D–COOH** Complex mass spectrum (left panel). Deconvoluted mass spectrum (right panel). Found: 30606.0, calculated for [C_1358_H_2097_N_367_O_426_S_7_]: 30604.8.

Preparation of GFP– GGGKDLEGKGGS[KHL]GSGS[KHL]GGSKYPYDVPDYAKD–COOH

Protein was prepared as described in the general method.

**ESI-MS characterization of protein conjugate sfGFP [KHL]** Complex mass spectrum (left panel). Deconvoluted mass spectrum (right panel). Found: 30916.5, calculated for [C_1376_H_2131_N_369_O_428_S_7_]: 30915.2.

Preparation of GFP– GGGKDLEGKGGSAGSGSAGGSKYPYDVPDYAKS–COOH

Protein was prepared as described in the general method.

**ESI-MS characterization of protein conjugate sfGFP–pep1 [A]–S–COOH** Complex mass spectrum (left panel). Deconvoluted mass spectrum (right panel). Found: 30577.5, calculated for [C_1357_H_2097_N_367_O_425_S_7_]: 30576.8.

Preparation of GFP– GGGKDLEGKGGSAGSGSAGGSKYPYDVPDYAKS–CONH_2_

Protein was prepared as described in the general method.

**ESI-MS characterization of protein conjugate sfGFP–pep1 [A]–S–CONH_2_** Complex mass spectrum (left panel). Deconvoluted mass spectrum (right panel). Found: 30576.5, calculated for [C_1357_H_2098_N_368_O_424_S_7_]: 30575.8.

Preparation of mCherry–GGGRRLEGKEEDEKGSRASDRFRGLR–COOH

Protein was prepared as described in the general method.

**ESI-MS characterization of protein conjugate mCherry–pep2 [RxxG]–R–COOH** Complex mass spectrum (left panel). Deconvoluted mass spectrum (right panel). A truncated species of mCherry with mass 22292.5 Da is observed. Initiator methionine processing is incomplete, providing two product mass peaks with 131.5 Da difference. Found: 30111.5, calculated for [C_1333_H_2064_N_366_O_413_S_9_]: 30113.36.

Preparation of mCherry–GGGRRLEGKEEDEKGSRASDDFRDLR–COOH

Protein was prepared as described in the general method.

**ESI-MS characterization of protein conjugate mCherry–pep2 [RxxG]–R–COOH** Complex mass spectrum (left panel). Deconvoluted mass spectrum (right panel). A truncated species of mCherry with mass 22310.0 Da is observed. Initiator methionine processing is incomplete, providing two product mass peaks with 131 Da difference. Found: 30129.5, calculated for [C_1333_H_2059_N_363_O_417_S_9_]: 30130.30.

Preparation of mCherry–GGGRRLEGKEEDEKGSRASDDFRDLR–CONH_2_

Protein was prepared as described in the general method.

**ESI-MS characterization of protein conjugate mCherry–pep2 [DxxD]–R–CONH_2_** Complex mass spectrum (left panel). Deconvoluted mass spectrum (right panel). A truncated species of mCherry with mass 22309.0 Da is observed. Initiator methionine processing is incomplete, providing two product mass peaks with 131.5 Da difference. Found: 30127.5, calculated for [C_1333_H_2060_N_364_O_416_S_9_]: 30129.31.

Preparation of mTAGBFP2–GGGRRLEGKEEDEKGSRASDRFRGLR–COOH

Protein was prepared as described in the general method.

**ESI-MS characterization of protein conjugate mTAGBFP2–pep2 [RxxG]–R–COOH** Complex mass spectrum (left panel). Deconvoluted mass spectrum (right panel). Initiator methionine processing is incomplete, providing two product mass peaks with 131.5 Da difference. Found: 30177.0, calculated for [C_1336_H_2081_N_369_O_407_S_11_]: 30176.68.

Preparation of mTAGBFP2–GGGRRLEGKEEDEKGSRASDDFRDLR–COOH

Protein was prepared as described in the general method.

**ESI-MS characterization of protein conjugate mTAGBFP2–pep2 [RxxG]–R–CONH_2_** Complex mass spectrum (left panel). Deconvoluted mass spectrum (right panel). Initiator methionine processing is incomplete, providing two product mass peaks with 130.5 Da difference. Found: 30193.5, calculated for [C_1336_H_2076_N_366_O_411_S_11_]: 30193.62.

Preparation of mTAGBFP2– GGGRRLEGKEEDEKGSRASDDFRDLR–CONH_2_

Protein was prepared as described in the general method.

**ESI-MS characterization of protein conjugate mTAGBFP2–pep2 [DxxD]–R–CONH_2_** Complex mass spectrum (left panel). Deconvoluted mass spectrum (right panel). Initiator methionine processing is incomplete, providing two product mass peaks with 130.5 Da difference. Found: 30193.0, calculated for [C_1336_H_2077_N_367_O_410_S_11_]: 30192.62.

### NMR spectra
