# Supplementary material for: C-terminal amides mark proteins for degradation via SCF–FBXO31
Source: Nature. 2025 Jan 29;638(8050):519–27. doi: 10.1038/s41586-024-08475-w (PMC11821526; doi:10.1038/s41586-024-08475-w)
Supplement: Supplementary file 2 — Reporting Summary [file 41586_2024_8475_MOESM2_ESM.pdf]

nature portfolio

Corresponding author(s): Jeffrey W. Bode; Jacob E. Corn
Last updated by author(s): Nov 7, 2024

Reporting Summary

Nature Portfolio wishes to improve the reproducibility of the work that we publish. This form provides structure for consistency and transparency in reporting. For further information on Nature Portfolio policies, see our Editorial Policies and the Editorial Policy Checklist.

Statistics

For all statistical analyses, confirm that the following items are present in the figure legend, table legend, main text, or Methods section.

n/a	Confirmed	
☐	☒	The exact sample size (*n*) for each experimental group/condition, given as a discrete number and unit of measurement
☐	☒	A statement on whether measurements were taken from distinct samples or whether the same sample was measured repeatedly
☐	☒	The statistical test(s) used AND whether they are one- or two-sided<br>*Only common tests should be described solely by name; describe more complex techniques in the Methods section.*
☒	☐	A description of all covariates tested
☐	☒	A description of any assumptions or corrections, such as tests of normality and adjustment for multiple comparisons
☐	☒	A full description of the statistical parameters including central tendency (e.g. means) or other basic estimates (e.g. regression coefficient) AND variation (e.g. standard deviation) or associated estimates of uncertainty (e.g. confidence intervals)
☒	☐	For null hypothesis testing, the test statistic (e.g. *F*, *t*, *r*) with confidence intervals, effect sizes, degrees of freedom and *P* value noted<br>*Give P values as exact values whenever suitable.*
☒	☐	For Bayesian analysis, information on the choice of priors and Markov chain Monte Carlo settings
☒	☐	For hierarchical and complex designs, identification of the appropriate level for tests and full reporting of outcomes
☒	☐	Estimates of effect sizes (e.g. Cohen's *d*, Pearson's *r*), indicating how they were calculated

Our web collection on statistics for biologists contains articles on many of the points above.

Software and code

Policy information about availability of computer code

Data collection	No specific software was used for data collection except the firmwares of measurement devices listed in Methods.
Data analysis	For FP-assays, curve fitting was performed using Prism 9 (GraphPad). CRISPR screen analysis was performed using MAGeCK (v0.5.9.3). Next generation sequencing validation of gene knockout was performed with CRISPResso2 (v2.3.0) TMT-based expression proteomics data was analyzed using Proteome Discoverer 2.4 (Thermo Fisher Scientific). For CRL-ABP DIA raw data was searched using DIA-NN (version 1.8.1) and the Perseus software package (v.1.6.7.0). IP-MS data was analyzed using FragPipe (v22.0) and the R package limma (3.58.1). Peptide library screens were analyzed using Mascot Server 2.8.1. (Matrix Science). Flow cytometry data was analyzed using FLowJo 10. Protein structures were visualized using ChimeraX-1.5. Paralogous protein sequences were aligned using JalView 2.11. RNA-seq data was analyzed with STAR-aligner (v 2.7.10a), FeatureCounts (v2.0.6) and DESeq2 (version 1.42.1).

For manuscripts utilizing custom algorithms or software that are central to the research but not yet described in published literature, software must be made available to editors and reviewers. We strongly encourage code deposition in a community repository (e.g. GitHub). See the Nature Portfolio guidelines for submitting code & software for further information.

Data

Policy information about availability of data

All manuscripts must include a data availability statement. This statement should provide the following information, where applicable:

- Accession codes, unique identifiers, or web links for publicly available datasets
- A description of any restrictions on data availability
- For clinical datasets or third party data, please ensure that the statement adheres to our policy

All large-scale data sets displayed in this paper are provided in extended data tables. All additional numerical data displayed in this study is provided as source data tables. Uncropped images of all gels and blots are provided in Supplementary Figure 1. Unprocessed data for proteomics and RNA-seq experiments is accessible through the following public repositories. Raw proteomics data is deposited alongside primary search results and complete search parameters at ebi.ac.uk/pride/ under accession numbers PXD055535 (FBXO31 IP-MS), PXD055814 (all CTAP identifications), PXD055518 (CRL profiling), and PXD055818 (TMT-based proteome quantification). Raw sequencing reads for RNA-seq samples are deposited to ncbi.nlm.nih.gov/sra under bioproject number PRJNA1173967. Deep sequencing read counts for CRISPR screen analysis are provided in Supplementary Table 9.
Public proteomics data re-analyzed in this study was obtained from ebi.ac.uk/pride/ under accession number PXD010154, sample numbers 1277, 1499, 1306 and 1296. Public protein structures were downloaded from www.rcsb.org (accession numbers 5VZU, 6TTU, 6DO3, 6LEY and 7Y3A). RNA-seq reads were aligned against the human genome reference GRCh38. Proteomics data was searched against the Uniprot human proteome reference UP000005640.

Research involving human participants, their data, or biological material

Policy information about studies with human participants or human data. See also policy information about sex, gender (identity/presentation), and sexual orientation and race, ethnicity and racism.

Reporting on sex and gender	n.a.
Reporting on race, ethnicity, or other socially relevant groupings	n.a.
Population characteristics	n.a.
Recruitment	n.a.
Ethics oversight	n.a.

Note that full information on the approval of the study protocol must also be provided in the manuscript.

Field-specific reporting

Please select the one below that is the best fit for your research. If you are not sure, read the appropriate sections before making your selection.

☒ Life sciences ☐ Behavioural & social sciences ☐ Ecological, evolutionary & environmental sciences

For a reference copy of the document with all sections, see nature.com/documents/nr-reporting-summary-flat.pdf

Life sciences study design

All studies must disclose on these points even when the disclosure is negative.

Sample size	For CRISPR screening, the chosen library representation, number of sgRNAs and replicates was based on prior experience with CRISPR screening and in accordance with mathematical models of screen performance (Nagy & Kampmann, BMC Bioinfrmatics 2017).<br><br>For IP-MS experiments, replicate numbers of initial experiments were based on prior experience. Based on the variation of initial experiments a sample size of n=3 was estimated to be required for detecting >2-fold enrichment with a power of >0.8 using a two-sided unpaired t-test comparing means of spectral counts. Based on these estimates, other IP-MS experiments with identical cell types and antibodies were also performed using three replicates.<br><br>All other experiments were performed on purified proteins and immortalized isogenic human cell lines that display minimal variance between independent experiments. We therefore chose a sample size of n=3 based on own experience and common practice in the field for work with immortalized cell lines.
Data exclusions	No data was excluded from analysis for any of the shown experiments. In-cell protein stability assays were aborted if electroporation failed.
Replication	Replicate numbers and designs of individual experiments are indicated in the respective figure legends. In addition, key experiments including in cell degradation assays, co-IP assays and in vitro binding assays were independently replicated by two or more experimenters. No attempt at replication failed.

Randomization	All experiments were performed on immortalized human cell lines or purified proteins. No assignment of specimens into experimental groups which would require randomization was performed.
Blinding	All measurements were performed automatically by devices (flow cytometers, photometers, gel scanners, mass spectrometers or NGS devices). No manual counting or measurements were performed. Therefore, no blinding was required.

Reporting for specific materials, systems and methods

We require information from authors about some types of materials, experimental systems and methods used in many studies. Here, indicate whether each material, system or method listed is relevant to your study. If you are not sure if a list item applies to your research, read the appropriate section before selecting a response.

Materials & experimental systems

n/a	Involved in the study
☐	☒ Antibodies
☐	☒ Eukaryotic cell lines
☒	☐ Palaeontology and archaeology
☒	☐ Animals and other organisms
☒	☐ Clinical data
☒	☐ Dual use research of concern
☒	☐ Plants

Methods

n/a	Involved in the study
☒	☐ ChIP-seq
☐	☒ Flow cytometry
☒	☐ MRI-based neuroimaging

Antibodies

Antibodies used	Primary western blotting antibodies: FBXO31 (Abcam, ab86137), FBXO31 (HPA HPA030150), GFP (Abcam, ab6556), mCherry (Abcam, ab183628), SKP1 (Cell Signaling Technology, 2156), CUL1 (Invitrogen, 71-8700), AARS1 (Fortis/Bethyl Life Science, A303-473A), GLUL (Fortis/Bethyl Life Science, A305-323A), Cyclin D1 (Abcam, ab134175), HA (Cell Signaling Technology, 3724) and SUGT1 (Bethyl, A302-944A). Primary western blotting antibodies for mCherry and GFP were used at 1:2000 dilution. All other primary antibodies were used at 1:1000 dilution.<br><br>Secondary western blotting antibodies: LI-COR Biosciences, cat. nr. 926-32213 and 926-68072. Dilution 1:15000<br><br>Flow cytometry antibodies: APC-anti-human-CD55[JS11] (Biolegend). Dilution 1:200 for cell immunostaining.
Validation	Antibodies for FBXO31 and HA were validated by overexpression in knockout backgrounds. Antibodies against GFP, mCherry and SUGT1 were validated by immuno-blotting of purified proteins. The SKP1 antibody was chosen based on the vendors' validation and 34 previously published studies. The CUL1 antibody was chosen based on 60 previous publications and validated internally by treatment with neddylation inhibitor MLN4924 nducing the expected Mw shift. The Cyclin D1 antibody was chosen based on previous validation by overexpression and studies of its regulation (e.g. doi.org/10.1038/s41586-021-03445-y). The AARS1 antibody was validated in previous studies by cDNA overexpression (e.g. doi.org/10.1101/2022.05.25.493316). The GLUL antibody was validated by the vendor by immunoprecipitation and blotting with two independently derived antibodies.

Eukaryotic cell lines

Policy information about cell lines and Sex and Gender in Research

Cell line source(s)	Parental cell lines (K562, HEK293, HEK293T) were obtained from ATCC or the Berkeley Cell Culture Facility (UC Berkeley). SF9 insect cells were obtained from Thermo Fisher Scientific. NPCs were derived by the group of Prof. Jessberger
Authentication	Parental cell lines were obtained authenticated from certified vendors. HEK293 and HEK293T were distinguished using custom primers targeting the large T antigen. K562 CRIPSRi cells were re-validated by STR-phenotyping (Microsynth). Neural stem cell identity and differentiation was confirmed by immunofluorescence imaging and transcriptomics. SF9 insect cells were obtained authenticated from Thermo Fisher.
Mycoplasma contamination	Human cell lines were regularly tested for mycoplasma contamination using a commercially available kit (Lonza, MycoAlert). Throughout the duration of this study, no contamination was detected. Sf9 cells used exclusively for protein production were not tested for presence of mycoplasma.
Commonly misidentified lines<br>(See ICLAC register)	none

Plants

Seed stocks	n.a.
Novel plant genotypes	n.a.
Authentication	n.a.

Flow Cytometry

Plots

Confirm that:

- [x] The axis labels state the marker and fluorochrome used (e.g. CD4-FITC).
- [x] The axis scales are clearly visible. Include numbers along axes only for bottom left plot of group (a 'group' is an analysis of identical markers).
- [ ] All plots are contour plots with outliers or pseudocolor plots.
- [x] A numerical value for number of cells or percentage (with statistics) is provided.

Methodology

Sample preparation

(See also Methods). For protein stability assays, cells were centrifuged and resuspended in PBS prior to acquisition. Cell-surface expression of CD55 was assessed by immunostaining (incubation with antibody APC-CD55 diluted 1:200 in PBS + 5% FBS, see Methods).

Instrument

Analytical flow cytometry was performed on an Attune NxT cytometer (Thermo Fisher Scientific). Cell sorting was performed using a Sony SH-800 cell sorter.

Software

FlowJo 10

Cell population abundance

For CRISPR screening, 32 million cells of a background population of GFP-negative cells (bottom 50%) were obtained per replicate. For the CTAP-clearance deficient target population (BFP-GFP+; ca), 1.3 and 2.2 million cells were obtained per replicate.

Gating strategy

See also Supplementary Figure 2. Events were subsetted for live cells based on their FSC/SSC characteristics and further gated for singlets based on height and width of the forward scatter. For competitive proliferation assays, cells expressing FBXO31 cDNAs were identified by additional gating for the co-delivered GFP marker, using non-transduced cells as a reference sample.

For CRIPSR screening, the CTAP degradation-proficient background population was defined as the lower 50% of GFP expression, which also appears GFP-negative compared to a non-nucleofected control population. The CTAP degradation-proficient target population was defined as BFP-GFP+ based on a negative control population receiving no fluorescent protein. For all other experiments, no dichotomization into marker-positive and -negative populations was performed.

We confirmed in initial experiments that protein electroporation uniformly delivered fluorescent reporter proteins to all cells, therefore not necessitating division into electroporated and non-electroporated cells. We therefore extracted the median fluorescence intensity for all live-gated single cells to quantify model substrate abundance throughout protein stability assays.

For dual-color reporter assays of protein stability, cells were gated for successful transduction and selection for the reporter vector by subsetting live single cells for mCherry-positive cells.

Where a single fluorescence parameter (GFP) or its derivative (dual-color-reporter) was analyzed, fluorescence levels were plotted as histograms instead of pseudocolor or contour plots.

- [x] Tick this box to confirm that a figure exemplifying the gating strategy is provided in the Supplementary Information.
