# Supplementary material for: C-terminal amides mark proteins for degradation via SCF–FBXO31
Source: Nature. 2025 Jan 29;638(8050):519–27. doi: 10.1038/s41586-024-08475-w (PMC11821526; doi:10.1038/s41586-024-08475-w)
Supplement: Supplementary file 4 — Supplementary Tables 1–9. [file 41586_2024_8475_MOESM4_ESM.zip › 2023-06-10327B-s4/Supplementary Table legends.docx]

Supplementary Table legends

Supplementary Table 1

Results of CRISPR screen for CTAP clearance factors including gene-level analysis of enrichment in CTAP clearance-deficient versus -proficient cells and raw sgRNA read counts.

Supplementary Table 2

List of peptides identified by MS from pooled peptide co-IP and input library. Scaled reporter ion intensities represent enrichment of peptides in IP versus input samples averaged across two parallel IP experiments.

Supplementary Table 3

List of all detected CTAP peptides.

Supplementary Table 4

List of proteins identified in CRL profiling in untreated versus H_2_O_2_-treated K562 cells.

Supplementary Table 5

List of proteins identified by HA-FBXO31 IP-MS from untreated or H_2_O_2_-treated HEK293T cells.

Supplementary Table 6

Differential gene expression results for RNA-seq samples of FBXO31 knockdown in HEK293T, NPCs and NPC-derived neurons.

Supplementary Table 7

List of proteins identified by HA-FBXO31(D334N) IP-MS from HEK293T cells.

Supplementary Table 8

Differential protein abundance following 12h induction of FBXO31 wildtype or D334N in HEK293T cells measured by whole proteome TMT-MS.

Supplementary Table 9

Peptides, plasmids and oligonucleotides used in this study.
